# Privatization of public goods: Evidence from the sanitation sector in Senegal^☆^

**DOI:** 10.1016/j.jdeveco.2022.102971

**Published:** 2023-01

**Authors:** Joshua W. Deutschmann, Jared Gars, Jean-François Houde, Molly Lipscomb, Laura Schechter

**Affiliations:** a University of Chicago, United States of America; b University of Florida, United States of America; c JILAEE, Argentina; d University of Wisconsin–Madison, United States of America; e University of Virginia, United States of America; f NBER, United States of America

**Keywords:** Sanitation, Privatization, Urban development

## Abstract

Privatization of a public good (the management of sewage treatment centers in Dakar, Senegal) leads to an increase in the productivity of downstream sewage dumping companies and a decrease in downstream prices of the services they provide to households. We use the universe of legal dumping of sanitation waste from May 2009 to May 2018 to show that legal dumping increased substantially following privatization—on average an increase of 74%, or an increase of about 1640 trips to treatment centers each month. This is due to increased productivity of all trucks, not just those associated with the company managing the privatized treatment centers. Household-level survey data shows that downstream prices of legal sanitary dumping decreased by 5% following privatization, and DHS data shows that diarrhea rates among children under five decreased in Dakar relative to secondary cities in Senegal following privatization with no similar effect on respiratory illness as a placebo.

## Highlights

• We study the privatization of sewage treatment centers in Dakar, Senegal.
• Privatization increases the productivity of sewage dumping companies by 74%.
• Prices paid by households for sanitation services fall by 5% following privatization.
• Diarrhea rates among children in Dakar fall relative to other cities in Senegal following privatization.

## 1 Introduction

Public utilities in developing countries are often poorly managed and fall into disrepair due to low state managerial capacity and poorly designed incentive systems. Since public utilities provide key services, the impact of poor management can be substantial. Upstream inefficiencies can raise prices and reduce supply downstream. One response to the difficulty of government management of utility services has been to privatize the services or operate them through public–private partnerships, potentially increasing efficiency and reducing costs.

Privatization may lead to improvement or deterioration in service provision. On the one hand, privatization can improve the efficiency of the utility and the quality of the services it provides if the government lacks the capacity to adequately manage it (Hart et al., 1997), or if the government has political objectives which lead to non-meritocratic hiring practices, corruption, or unsustainably low pricing (Galiani, 2022). On the other hand, private companies may take advantage of the natural monopoly and ignore health externalities, raising prices (Chong and de Silanes, 2004, Megginson and Netter, 2001). Because it is not possible for the government to contract on all aspects of service provision, privatization may result in lower quality or less extensive service provision. Incentives may be further impacted by the government’s inability to credibly commit to privatization beyond a relatively short time period (Galiani, 2022).

When the acquiring firm is already operating in the industry, privatization also allows that firm to gain direct access to a key input. Vertical integration can lead to efficiency gains, for example by increasing the incentive of the operator to invest in higher service quality or reduce prices. However, to the extent that firms have market power, a vertically integrated operator can also fully or partially prevent or “foreclose” access to the utility for competing firms, for example by raising the access price or reducing quality. The empirical evidence on the net effect of vertical integration is mixed. While some papers have confirmed the presence of foreclosure effects by raising rivals’ costs (Chipty, 2001, Luco and Marshall, 2020), the general view of antitrust authorities is that efficiency effects tend to dominate (Hortaçsu and Syverson, 2007).

We measure the overall effect of privatization of sewage treatment centers in Dakar, Senegal in November 2013, focusing on the effects on productivity, downstream prices, and health. Outcomes for downstream firms include the intensity of capital use, the propensity to invest in new capital, the territory in which companies work, the number of days they operate in a month, and whether they enter or exit the market. Outcomes for households include the prices paid for downstream services and the incidence of diarrheal disease. Before privatization, the treatment centers were managed by ONAS (the Senegalese National Sanitation Office), a government agency charged with managing Senegal’s sanitation sector. Trucks drive to the sewage treatment centers to dump waste which they pumped out of residential latrine pits and septic tanks. The treatment centers charge trucks a fee for dumping. The centers then process the waste in holding ponds with some filtration. This is the only legal (and hygienic) form of disposal of sewage waste in Dakar for households without a direct connection to a sewerage system.

In 2012, following complaints by the truckers about long wait times and frequent closures at the treatment centers and encouragement from the Gates Foundation, the Senegalese government decided to privatize their management.^1^ They ran a call for bids and selected the company Delvic Sanitation Initiatives, formed from a partnership of two of the largest companies in the sanitation truck sector (Delta and Vicas), to take over management of the centers in 2013 via a public–private partnership.

There are several unique aspects to this context that make it a particularly interesting environment in which to study the impact of privatization. First, most studies measuring the impact of privatization look at industries such as water, electricity, and telecommunications which interact directly with consumers (McKenzie and Mookherjee, 2003) and focus on the poor incentives that utilities may have in terms of reaching the ‘last mile’ (Ashraf et al., 2016) or investing in maintenance of the network (McRae, 2015). In our study, the treatment centers do not directly serve consumers but instead are an upstream input into the production of sanitation services. Sewage treatment center management affects the efficiency of trucking companies, which in turn supply sanitation services to consumers downstream. Second, it is rare to have data from before and after privatization on downstream businesses. In this case, the treatment centers collected the license plate of each truck that dumped waste at the center both before and after privatization. We collected and digitized the records from 2009 through 2018. From this data, we can observe how privatization affected truckers. Third, the fee for using these centers is fixed by the government and remained unchanged after privatization, allowing us to study the impacts of privatization absent any price effects discussed above. This allows us to unbundle the impacts of different aspects of privatization, although inasmuch as this type of fixed price after privatization is less common it may decrease the external validity of this study. Finally, most of the literature measuring the impacts of privatization has focused first on Eastern Europe (Barberis et al., 1996, Megginson and Netter, 2001) and later on Latin America (Chong and de Silanes, 2004, Granados and Sánchez, 2014, McKenzie and Mookherjee, 2003, Saiani and de Azevedo, 2018). We study privatization in Africa where there is much less evidence (Kirkpatrick et al., 2006, Kosec, 2014). To our knowledge, this case represents the first study of privatization of sewage treatment centers in Sub-Saharan Africa.

Identification is difficult because privatization occurred at all three treatment centers at the same time. As a result, the primary identification strategy in this paper is an event study. However, we are able to combine several large data sources and evaluate differential impacts of the privatization on various market players using difference-in-differences analysis. We have data on the universe of legal dumping in Dakar over a period of nine years, with the privatization occurring towards the middle of that period, so we are able to see the extent to which the privatization led to temporary versus permanent changes in Dakar’s sanitation market. Our data allows us to observe dumping at the truck and company level (on average companies own 2 trucks).

Sanitation companies rely on the treatment centers as part of their supply chain: they need to dump waste at a center between each job, so any blockages at the center level can have substantial impacts on their productivity. Volume at the centers increased substantially following privatization. We see an average increase of 1,644 trips per month from an initial average prior to privatization of 2,224 trips per month.

Prior to privatization, the treatment centers were managed by ONAS, the government agency responsible for sanitation in Senegal, but privatization meant that management of the treatment centers was delegated to a partnership between two sanitation companies. Privatization led to large increases in the productivity of truckers downstream. The average company did 51% more jobs following privatization. The productivity increases were not limited to the companies managing the privatized centers. In fact, if anything, the effects on those two companies were more muted. The increased productivity per truck is therefore not driven by these two vertically integrated downstream companies.

We show that the large productivity changes at the company level are due to increased truck-level productivity, increased investment in capital stock, improved management of capital, and changed hours of operations. The highly disaggregated nature of our data (at the truck-dumping level) allows us to estimate the change in activity at the truck level and the extent to which trucks work on weekends. We find that most companies not affiliated with Delvic do not invest in additional trucks following privatization, but their trucks are 12 percentage points more likely be active in a given month. This is an important source of gains in productivity: at baseline, the average company had only 76% of their fleet actively dumping in any given month.^2^ Maintenance issues are often expensive, and parts needed for repair can take time to source, so this is likely due to better continued management of their fleet. Trucks do approximately 58% more trips per month following privatization. Trucks work 3.8 additional days per month on average, work 0.5 more Saturdays and 0.4 more Sundays in a month, and visit more treatment centers per month on average.

Improving the efficiency of the sanitation sector is especially important due to its direct connection with diarrheal diseases, which have important welfare impacts on communities and lasting impacts on children (Hammer and Spears, 2016). Diarrheal diseases remain a major public health problem in African cities (WHO, 2017), due in part to rapid urbanization without sufficient investment in sanitation infrastructure. In Senegal, a country where both large and secondary cities struggle to provide basic services, diarrhea is the leading cause of death among children under the age of 5, responsible for 14% of total disability-adjusted life years (Wang et al., 2016). Notably, the fraction of Senegal’s population living in urban areas has more than doubled since 1960 and reached 48% in 2020 (United Nations, 2019), placing more pressure on the sanitation infrastructure.

A household whose latrine or septic tank has filled with waste has two choices to desludge it (i.e., to remove the waste). In our setting, they must do this once or twice a year. They can hire a person to manually shovel the waste out of the pit and leave it on the street or in an open field nearby (a manual desludging), or they can hire a trucker to pump the waste and take it away in a truck (a mechanized desludging). In the case of a mechanized desludging, the trucker can dump the sewage illegally in nearby canals, vacant lots, or in the ocean, or the trucker can dump the sewage legally at a treatment center. Low truck productivity therefore results in high downstream prices of mechanized desludgings and substitution toward less sanitary manual desludgings, which leads to substantial and enduring health impacts.

Increasing the amount of waste disposed in treatment centers has important impacts on health.^3^ The large increase in sanitary dumping of waste at treatment centers that we measure post-privatization would have had a substantial impact on quality of life. We use DHS data to compare the incidence of diarrhea in children under five in Dakar with the incidence in secondary Senegalese cities of similar population density prior to and post privatization. Following privatization, diarrhea rates among children under five decreased in Dakar relative to other Senegalese cities which did not have similar sanitation reforms at the time, suggesting that the increased use of the privatized treatment centers improved health. A similar effect is not seen for respiratory illness which would not be expected to be significantly affected by improvements in sanitation.^4^

[18] suggest that outsourcing service provision to private providers can improve quality and reduce costs, but this may crucially depend on the ability of a service provider to affect quality along non-contracted dimensions. Existing work studying the outsourcing of service delivery in a variety of settings (Banerjee et al., 2019, Bloom et al., 2006, Loevinsohn and Harding, 2005, Romero et al., 2020) underscores the importance of quality observability and enforcement. We study a setting where quality of service provision is observable to end-users (sanitation truckers), along dimensions such as wait times, facility reliability, and operating hours. We provide evidence that privatizing upstream sanitation services resulted in improved downstream service delivery without reductions in observable quality.

Most previous studies of the privatization of sanitation services involve the privatization of water and sewerage systems. Almost all such studies find privatization leads to increases in water and sewerage connections (Chong and de Silanes, 2004, McKenzie and Mookherjee, 2003), although Kirkpatrick et al. (2006) find no effect of water privatization in Africa. Some studies go one step further to look at effects on health and again most find improvements in health outcomes, especially in the poorest areas or among the poorest individuals (Galiani et al., 2005, Kosec, 2014, Saiani and de Azevedo, 2018), with one negative effect found by Granados and Sánchez (2014). The existing literature shows the positive impacts of privatization of sanitation utilities that provide piped water or sewerage service directly to consumers. This is the only paper we know of looking at the privatization of sanitation in the form of an upstream input: sewage treatment centers.

Despite the lack of evidence on the privatization of sewage treatment centers, these centers are extremely important. According to the World Bank Africa WSS Utility Survey (World Bank, 2005), among low income (non-fragile) countries in Africa only 26.7 percent of the population had a wastewater connection, 85 percent of wastewater treatment plants were non-functioning, and only 8.5 percent of the wastewater collected in the existing service area was subject to any form of treatment as of 2005. A substantial majority of sewage waste in Africa is collected outside of the piped sewerage network, and must therefore use trucking providers like those we study in this paper. Improving the productivity of these trucks through increasing the managerial capacity and functionality of treatment centers could therefore create significant welfare benefits.

## 2 Background

Dakar has three sewage treatment centers in the Rufisque, Niayes, and Camberene neighborhoods, all of which opened between 2006 and 2008. The treatment centers collect all of the legally deposited waste in the city and there are no other legal locations for trucks to dump sludge in the Dakar area.^5^ These treatment centers collect and process waste produced by households without a connection to the sewage network, which included more than 75% of the city’s population in 2013 (Sene, 2017). The centers originally charged truckers 200 CFA (or 40 cents) per cubic meter of waste dumped, and the modal truck holds eight cubic meters of waste. This fee was raised to 300 CFA in early 2010.

Prior to the 2013 privatization, vacuum truck operators complained about the state of the three treatment centers. There were typically long lines of trucks waiting to dump as the dumping process was slow; treatment centers were closed on weekends and often closed early in the afternoon; and one of the centers closed multiple times, sometimes for months at a time, because it was either overwhelmed with sludge or its equipment had broken down.^6^ These disruptions restricted the number of jobs that truckers were able to do during a day, especially the number of jobs for which they dumped the sludge legally. Truckers rely on being able to dump their sludge in a timely fashion and continue on to other jobs. Perhaps as a result of these difficulties, illegal dumping was common, with one report estimating that in 2008 about 50% of waste collected was dumped outside of legal dumping sites (Gold, 2014).

In late 2011, the Senegalese government launched an ambitious program to restructure the market for sanitation services in Dakar, the “Faecal Sludge Market Structuring Programme” (PSMBV). With technical and financial support from the Bill & Melinda Gates Foundation, the program had a particular focus on involving the private sector in all facets of sanitation service provision for households (Diop and Mbeguere, 2017, Mbeguere et al., 2011). As one component of this program, the government launched a call for proposals for a private enterprise to take over the management of the treatment centers. The winning bid was submitted by Delvic, a new partnership formed between two of the largest waste removal companies in the city (Delta and Vicas).^7^ The Delvic partnership officially began managing the treatment centers in November 2013. This privatization process did not give Delvic ownership of the treatment centers, and major investments in facilities and equipment remained the responsibility of the National Sanitation Office of Senegal (ONAS). Fees charged to truckers to use the treatment centers remained fixed by government regulation and did not change after privatization. The privatization did give Delvic full authority to manage the centers as well as 50% of the net revenue collected from the centers after they had paid ONAS an annual licensing fee. This type of partnership is called an affermage contract, a model of privatization somewhat common in former French colonies (Janssens et al., 2011). The private company runs the utility and receives a set portion of the receipts as payment for running the utility, but the government remains the owner of the capital. In return, Delvic was required to make small investments necessary for the operation of the facilities and to ensure that all users had access to the facilities. The annual operating profits of the centers increased from $7,100 prior to privatization to $33,300 in 2016 (Diop and Mbeguere, 2017). In 2013 and 2014, ONAS reportedly earned 150%–170% of their pre-privatization 2012 revenue from the revenue-sharing arrangement with Delvic (Sene, 2017).

According to truck operators surveyed at the time, following privatization there were fewer disruptions to service at the centers and centers were better maintained. Desludging trucks were able to get in and out of the centers faster as they added dumping capacity and reduced wait times (which had commonly been over an hour before privatization). In addition, some centers were open longer hours and on weekends. Finally, truckers appreciated that the centers made restrooms available as they had few other options for places to stop during work hours. Appendix Table A1 provides an overview of self-reported changes that truck operators noted at the treatment centers and how they adjusted their behavior following privatization. More than three quarters of the truckers think the post-privatization changes have been positive, with longer opening hours and more days open topping the list of positive changes. For the quarter who think privatization led to negative changes, the most common complaint is increased dumping costs. While the official dumping cost was not increased following privatization, it is possible that treatment center operators use discretion in terms of when and how to collect payments. Four-fifths of the truckers state that they work more days and/or longer hours after privatization. Prior to privatization, the mean truck completed just 19.4 jobs per month conditional on doing at least one job. After privatization, this increased by 55% to 30.1 jobs per month, suggesting many trucks were operating substantially below full capacity.

**Fig. 1:**
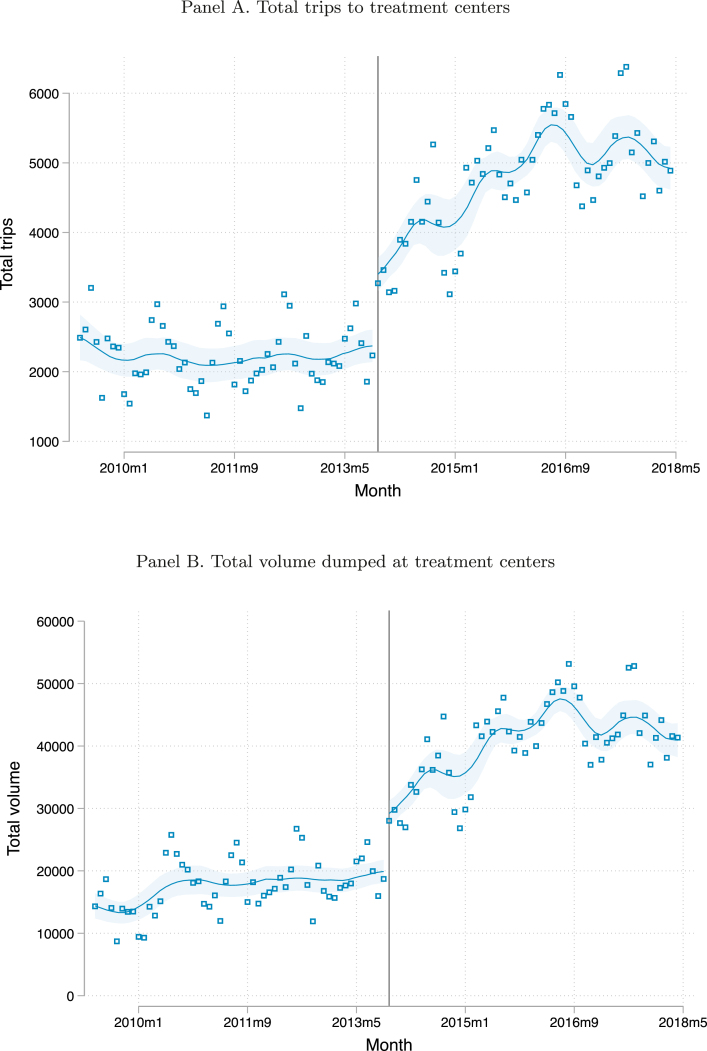
Aggregate activity at the treatment centers in each month. Note: Locally-weighted polynomial regressions of the total number of trips and total volume dumped at the month level between May 2009 and May 2018. The y-axis in panel A shows the number of trips made to all treatment centers in each month, while the y-axis in panel B shows the total volume of waste dumped (in $m^{3}$) at all treatment centers as measured by the capacity of the trucks visiting, not the actual volume of sludge deposited. The solid vertical line indicates the month of privatization (November 2013). The shaded area indicates 95% confidence bands.

**Fig. 2:**
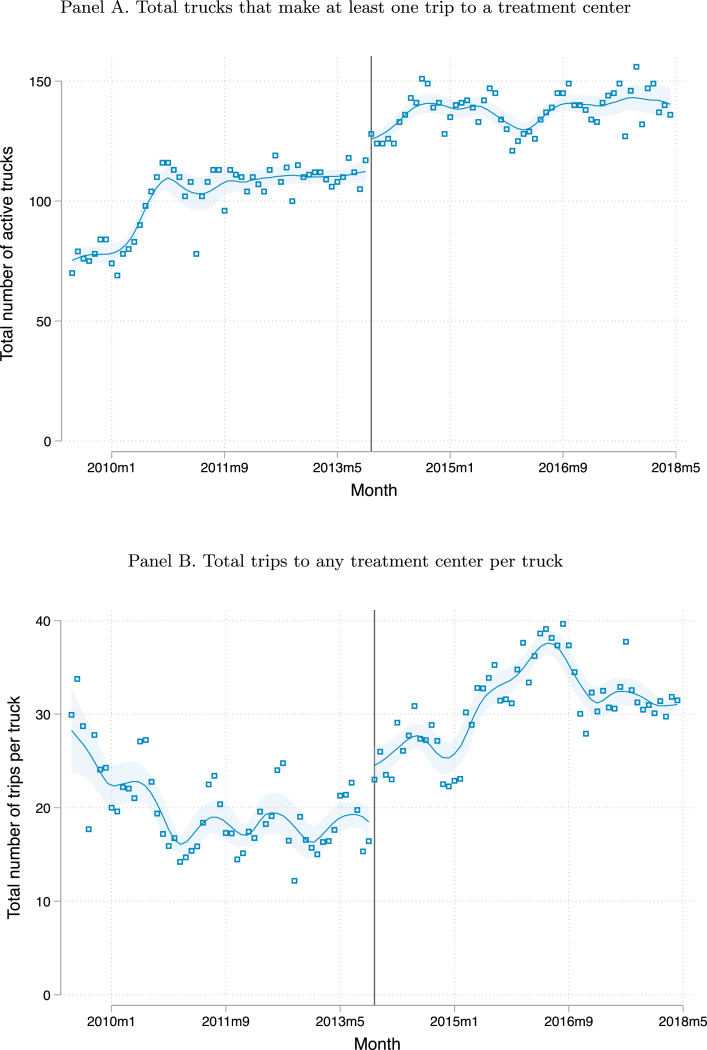
Truck-level activity at the treatment centers in each month. Note: Locally-weighted polynomial regressions of productivity measures at the month level between May 2009 and May 2018. The y-axis in panel A shows the number of trucks that made at least one trip to any treatment center in each month, while the y-axis in panel B shows the number of trips per truck that made at least one trip to any treatment center in each month. The solid vertical line indicates the month of privatization (November 2013). The shaded area indicates 95% confidence bands.

The overall impact of privatization on the amount of dumping done at the three treatment centers is shown in Fig. 1, Fig. 2. Fig. 1 shows that the total number of dumping trips made and the total volume dumped were quite flat between 2009 and 2013 before privatization. Immediately following privatization, there is a sharp increase in both trips and volume, with a continued increase thereafter until they plateau in 2017. Panel A of Fig. 2 shows that there was an upward trend in the number of trucks active in the market in 2010, after which it platteaued, with a discontinuity when privatization took place. Panel B of Fig. 2 shows a large increase in the intensity of truck usage after privatization. The number of trips per truck per month declined in 2010 and then platteaued prior to privatization.^8^ After privatization there was a discrete increase in the intensity of use of trucks followed by a continued increase.

This increase in dumping at the treatment centers may be due to an increase in households’ use of mechanized desludging and a decrease in their use of manual desludging. Almost all (98.9%) of the sludge dumped at the treatment centers is categorized as coming from a residence, while only 1% is categorized as coming from industry and 0.1% is categorized as coming from government offices.

The increase in dumping at treatment centers could also be due to a decrease in illegal dumping by trucks. Trucks may dump sewage in nearby sewage drains and canals of which there are a network around Dakar, directly into the ocean, at Yarakh (an unimproved designated dump site which officially closed when the treatment centers opened), or in vacant lots. Dumping at any place other than a treatment center is illegal and carries a substantial fine. According to discussions with the truckers, the threat of being caught is large. The fine for illegal dumping varies between $400 and $1,200 (the cost of approximately 16 to 48 household desludgings performed by a truck), though an offending trucker would typically offer a bribe rather than paying the full fine. The welfare impacts of truckers’ substituting from illegal dumping to dumping at the treatment centers may be similar to the welfare impacts of households’ substituting between manual and mechanized desludging, as illegally dumped sewage may be transported out of the immediate neighborhood of the household, but may still end up dumped close to communities. The increase in the overall amount of waste dumped at treatment centers is therefore a fair estimate of the welfare effects from the privatization policy.

### 2.1 Other interventions in the sanitation sector and other potential confounders

Privatization of the treatment centers was only one component of the larger PSMBV program that ONAS launched in 2011. One may be concerned that the impacts we attribute to privatization are confounded with the impacts of other activities related to this larger program. Because we were actively involved in a number of these projects for other research studies, we are able to estimate the maximum impact that each could have had on dumping at the treatment centers. We discuss the key components of the larger ONAS program in order to quantify how much of the effect we identify could have come from the other projects.

Overall, even under our most conservative calculations these projects could only explain a small share of the increase in desludgings, and the timing of most of these projects is months after the privatization so cannot explain the immediate large increase we see in Fig. 1. Only the first intervention we mention, the desludging call center, could explain at most 11% of the increase in volume, while the other interventions we describe can explain much less than that. Appendix Table A2 shows a regression version of the results in the figure. In addition to the full time period, we restrict the post-privatization period to be nine, six, and two months after November 2013. The impact on log(trips) in the full sample is 0.55, and declines to 0.52, 0.54, and 0.47 (all statistically significant), respectively when limiting the post-privatization time period to the shorter windows. Thus, the effect of privatization is 85 percent of the total effect within the first two months. This provides evidence that a large share of our estimated effect of privatization on trips is not due to the other programs that occurred in 2014. Appendix Table A3 shows that this aggregate increase in trips occurred at all three treatment centers.

#### Desludging call center.

In February 2014, ONAS launched a call center to connect households to mechanized desludging operators using auctions (Deutschmann et al., 2021). Even during the period when the call center was most highly advertised, volume at the call center never exceeded 200 auctions per month. The call center can therefore explain no more than 11% of the increase in volume at the treatment center (under the most conservative assumption that all households purchasing a mechanized desludging through the call center would have otherwise used a manual desludging).

#### Subsidies and mobile money saving program.

As part of a companion research project, we offered subsidies of different levels to 4100 households starting in late March 2014 to encourage them to sign up to purchase a mechanized desludging, and offered a mobile money savings program to some of the households who accepted our subsidy offer (Deutschmann et al., 2022, Lipscomb and Schechter, 2018). There were 348 subsidized mechanized desludgings purchased by 278 households through this intervention. Even with our most conservative calculation that none of these households would have purchased an unsubsidized mechanized desludging without our program, these additional 348 mechanized desludgings between March 2014 and May 2015 could account for at most 1% of the total increase in desludgings following privatization.^9^

#### Loan guarantee program.

As part of the PSMBV program, ONAS established a loan guarantee fund in partnership with the National Bank of Economic Development and the Association of Sanitation Actors of Senegal. This provided a financing mechanism for sanitation companies to invest in new or used trucks to be imported from Europe. The program resulted in 29 trucks entering service in Dakar, with the first trucks arriving in September 2014 (Sene, 2017). Given that privatization occurred in November of 2013, the timing is off to explain the increase in dumping in the months after privatization. In addition, while this could explain an increase in the number of trucks found in our analysis, our regressions only show a small and insignificant impact of the privatization on the number of trucks in the market. The companies most connected to Delvic (namely Delta and Vicas) add approximately three trucks after privatization, but this capital investment cannot be directly attributed to the privatization policy.

#### Other potential confounders.

One potential confound is that the accuracy of the documentation of the quantities of sludge arriving at treatment centers may change due to privatization. There are logistical processes in place to minimize misreporting, and little evidence that it took place, as discussed in more detail in the next section. It is also possible that privatization came with an increase in enforcement against illegal dumping and/or manual desludging. However, dumping outside of the treatment centers was illegal long before privatization and in our conversations with truckers and policy makers we did not hear any discussion of changes in enforcement.

It is important to note that we estimate the total effect (direct plus indirect) of privatization rather than simply the direct effect. The improvements we see in child health may capture both direct and indirect effects. Households may witness the improvement in local sanitation directly caused by privatization and react by making further improvements in their own sanitation choices (e.g., better latrines or separated animals). To the extent that privatization led to changes in household behavior, the total impact of privatization is the combination of the direct effect of the privatization of management of the centers as well as any additional indirect effects that came as a reaction to it.

Other potential confounding factors include population or political changes at the same time as privatization. We are not aware of any events which could have caused such a change. The outbreak of war in Mali in 2012 and instability following elections in The Gambia in 2017 may have caused temporary immigrant inflows to Senegal, but those events neither coincide in time with the privatization nor did they result in large or lasting changes in Senegal’s population. Dakar had the same mayor from 2009 to 2018, and Senegal has had the same president from 2012 through the present (2022). The new national government may have been more amenable to privatization, but to our knowledge the launch of the government’s sanitation program began before the presidential election and was not impacted by it.

## 3 Data

### Treatment center data.

Trucks that dump at one of the three treatment centers in Dakar are charged based on the size of their truck under the assumption that the truck is full. The treatment center manager writes a receipt for the dumping fee, and at the same time records basic data including the date, the license plate, and the size of the truck (in cubic meters) in a notebook. The centers did not change their record-keeping process in the year after privatization, only adopting a new computerized data entry system in late 2015. We collected all available records from the three treatment centers in Dakar from May 2009 through October 2018.

Many of the records were handwritten, which we then digitized, and this poses some problems. The ‘name’ field may show the owner’s name, the company’s formally registered name, the company’s informally used name, or the driver’s name. License plates are sometimes only partially recorded and numbers may be transposed. In addition, license plates are periodically changed, so the same truck may have multiple plates over the years of our data. To the extent possible, we correct license plates and assign the correct company name to each truck. Due to difficulties in cleaning the names and plates, the data contains more license plates and more companies than actually exist. This issue should not be affected by privatization and our company- and truck-level regressions will include either company or license plate fixed effects.

One might be concerned that Delvic, the company selected to manage the privatized treatment centers, would have an incentive to overstate volumes in order to appear particularly efficient and well-run. On the other hand, they might have an incentive to understate volumes so that they do not have to return as much money to the government. To explore this concern, in 2014 we sent two enumerators to each treatment center for three days to keep their own logs. The enumerator logs and the Delvic logs included the same number of dumps. A second concern may be that the managers behaved differently when the enumerators were there, but the volume reported on the days that the enumerators were there were very similar to the volume reported on other days in the same month without enumerator presence. In addition, for several time periods before and after privatization, we observe two distinct data sources: entry logs and payment registers. These are kept by different people, at different locations within the station, and they are handwritten, which would make coordinated misreporting difficult. These two data sources rarely show large discrepancies for a given day, and one does not show systematically greater volume than the other.

In late 2015, the record-keeping procedure at the treatment centers was changed. All three centers transitioned to a new digital platform for recording data. Previously, from 2012 to 2015 the Camberene center recorded visits in an Excel file. The other centers recorded visits on paper before 2015, as did Camberene until 2012. This change affected our ability to match plate and company names between the old and new data. This may cause us to overstate the number of companies in the data, but would not lead to additional jobs.

**Fig. 3:**
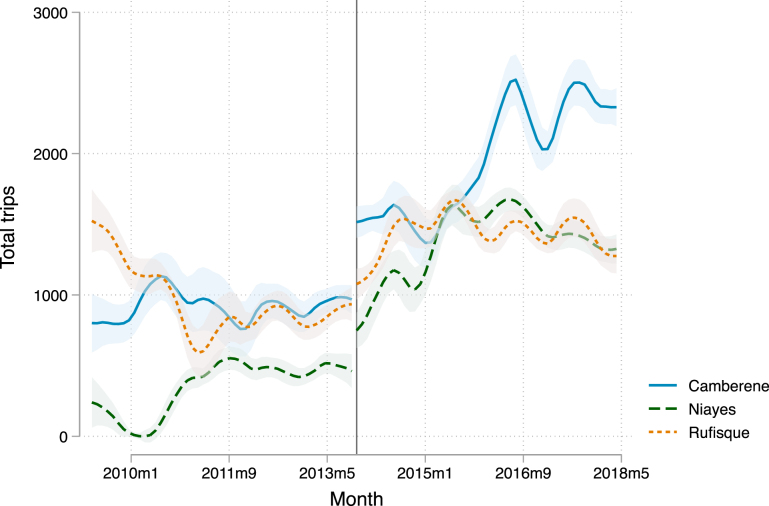
Trips to each treatment center in each month. Note: Locally-weighted polynomial regressions of trips to each center at the month level between May 2009 and May 2018. The y-axis shows the number of trips made to each treatment center in each month. The solid vertical line indicates the month of privatization (November 2013). The shaded area indicates 95% confidence bands.

We can evaluate the potential impact that this change in record keeping had on trips at the centers using Fig. 3. First, Camberene switched to digital record keeping in 2012. Fig. 3 shows a flat trajectory of trips for Camberene from late 2011 through mid 2013, suggesting that it is unlikely that digital record keeping impacted recorded volume. In addition, if digital record keeping made it easier for centers to record more trips following privatization, we would see a larger increase in trips in late 2015 for the Niayes and Rufisque centers than for Camberene. In fact, the opposite is true.

In addition to understanding the impacts of privatization on capital, capital use, productivity, and working hours of trucks, we are also interested in measuring whether privatization affects the territory in which each trucker works as this may impact the competitiveness of the market. To do so, we consider the number of treatment centers a truck visits in a given month. If privatization improves the reliable availability of treatment centers, truckers may be more willing and able to compete for business in a larger area.

### Household survey data.

We evaluate the downstream impact of privatization using household survey data measuring the date of the household’s most recent desludging, the price they paid for the desludging, and whether it was manual or mechanized. In order to control for local differences in wealth, accessibility, and soil type, we include subzone fixed effects.^10^ The sample is representative of the peri-urban Dakar population that is not connected to the sewer network. In total, there are 28 subzones and we survey an average of 127 households per subzone. The surveys took place in Sept–Dec 2012, Feb–May 2014, Jun–Jul 2014, and Mar–Jun 2015.

Households report the details of their most recent desludging, including the price, number of trips it took to complete, and whether it was mechanized or manual. For manual desludgings, the survey further distinguishes between paid manual desludgings (performed by a *baay pell*) and unpaid desludgings performed by a family member. We ask how long ago the household got its most recent desludging. The bunching of observations at 0 (this month), 3, 6, and 12 months ago suggests that this will not give us precise data on the exact timing of purchases but should give a general sense. We only consider desludgings purchased in the past 12 months to reduce recall bias. Our dependent variables of interest in these regressions are (i) whether the household purchased a mechanized desludging given that they desludged their pit, and (ii) the price they report paying for a desludging if they get a mechanized desludging. On average, 55% of desludgings are mechanized and the average price of a mechanized desludging is 23,286 CFA, or approximately $46.

### Household health data.

We estimate the impacts of privatization of waste treatment centers on health outcomes using eight rounds of Demographic Health Surveys (DHS) collected between 2005 and 2019 (Agence Nationale de la Statistique et de la Demographie-ANSD/Senegal, et ICF, 2019). We have data on children from a total of 6,828 households in 2005, 2010, 2012, 2014, 2015, 2016, 2017, 2018, and 2019. We consider two indicators of child illness — diarrhea incidence and cough incidence. We measure whether any child in the household under the age of 5 experienced an episode of illness in the two weeks prior to the survey.

The DHS is a nationally-representative repeated cross-section that is stratified by region and by urban vs rural. There are a total of 28 survey strata across 14 regions (one urban and one rural per region). The sample was drawn in two stages. First, within each strata, clusters (census districts) were drawn with probability proportional to the population of the cluster. The number of clusters varies by survey round between 200 and 400. In the second stage, 21 households were drawn from each cluster with equal probability.

In our analysis, we compare disease incidence in Dakar to disease incidence in other areas in Senegal based on two definitions of the control sample. The first definition that we use is *Urban* which comes from the DHS classification. This classification is based on the official definition of the National Statistical Office and includes 165 communes in Senegal with at least 10,000 inhabitants. The DHS sample does not include information on the specific commune from which an urban cluster is drawn, simply indicating whether they are urban. To construct a control group that is more similar to Dakar in terms of density and population, our second definition (*Cities*) uses the GPS coordinates of clusters to identify only those that are located in one of the seven largest cities in Senegal.^11^ Appendix Table A4 provides an overview of the number of households and clusters by survey round and definition. In 2012, for example, there are 153 households in 16 clusters in Dakar. The *Urban* definition includes 799 households in 63 clusters, while the *Cities* definition includes 452 households in 25 clusters.

### Summary statistics.

Table 1 provides summary statistics prior to privatization. We observe 97 companies in the data (the regressions are limited to the sample of companies present prior to privatization), owning an average of 2 trucks with 1.6 (80%) trucks active in an average month. Most (66%) companies are independent owner-operators with a single truck, while 28% are mid-sized (owning 2–5 trucks), and only 6% of companies own more than 5 trucks. Delta and Vicas, the two companies that partnered to form Delvic which manages the privatized treatment center, represent two out of the 97 companies.

**Table 1:** Summary statistics (pre-privatization)

Variable	(1)	(2)	(3)	(4)	(5)
	Obs	Mean	Std. Dev.	Min	Max
***Panel A: Company characteristics***					
Trucks owned	97	1.99	1.98	1	12
Share trucks active	97	0.80	0.20	0	1
Own 1 truck	97	0.66	0.48	0	1
Own 2-5 trucks	97	0.28	0.45	0	1
Own 5+ trucks	97	0.06	0.24	0	1
Delvic	97	0.02	0.14	0	1

***Panel B: Company level data (Monthly)***					
P(Active)	3489	0.85	0.36	0	1
Ln(Trips)	2971	2.83	1.25	0	7
Ln(Volume)	2971	4.94	1.25	1	9
Ln(Trucks owned)	3489	0.42	0.66	0	2
Trips	2971	35.39	57.34	1	718
Trips/truck	2971	18.18	16.00	1	103
Volume	2971	291.30	451.13	4	5591
Trucks owned	3489	2.02	1.95	1	12

***Panel C: Truck level data (Monthly)***					
P(Active)	7031	0.77	0.42	0	1
Ln(Trips)	5412	2.45	1.13	0	5
Ln(Volume)	5412	4.58	1.11	1	7
Trips	5412	19.43	18.81	1	143
Volume	5412	159.91	150.22	2	1000
Days worked	5412	9.35	6.17	1	26
Work Sat	5412	0.65	1.06	0	4
Work Sun	5412	0.06	0.27	0	4
N stations	4778	1.54	0.67	1	3

***Panel D: Household Data (Desludgings)***					
Pr(Mechanized)	3392	0.52	0.50	0	1
Price per trip (CFA)	1872	23285.64	7954.71	10000	50000
Ln(Price per trip)	1872	9.99	0.36	9	11

***Panel E: Household Data (Health)***					
Diarrhea (Dakar)	844	0.36	0.48	0	1
Diarrhea (Other urban)	3610	0.27	0.44	0	1
Diarrhea (Other cities)	2484	0.27	0.44	0	1
Cough (Dakar)	844	0.41	0.49	0	1
Cough (Other urban)	3610	0.31	0.46	0	1
Cough (Other cities)	2484	0.31	0.46	0	1

Note: Company and truck level variables (Panels A–C) are based on transaction data from all dumpings reported at treatment centers in Dakar between May 2009 and November 2013 (pre-privatization). The household data in Panel D includes retroactive desludging observations that took place between October 2011 and November 2013. The sample includes each household’s most recent desludging if it took place both in the year before the survey and prior to privatization. The price of mechanized desludging is winsorized at the bottom at 10,000 CFA and at the top at 50,000 CFA. Summary statistics for the household health data are calculated using three rounds of the Demographic and Health Survey (2005, 2010, and 2012).

Companies do an average of 35 (and trucks an average of 19) trips in months in which they do at least one trip. The fact that trucks can do from a minimum of 1 trip in a month to a maximum of 143 suggests that many trucks are not close to their capacity constraints. We see that 77% of trucks are ‘active’ (having made at least one trip to a treatment center) in an average month but they only work an average of 9.4 days per month. However, there are trucks that worked 26 days in a month. Trucks worked less than one Saturday per month and almost never worked on Sundays prior to privatization in months in which they dumped at least once.

Truck operators commonly favor a specific territory. The average trucker visits the same center for 92% of their monthly trips and goes to 1.54 treatment centers in a month. The treatment center that received the most visits prior to privatization was Camberene (45%), followed by Rufisque (32%) and Niayes (23%). Fig. 3 shows the total number of monthly trips to each treatment center. The share of truckers that favored each center is roughly equivalent to the total share of trips that each center receives. This may reduce travel costs, but may also mean that some areas are less competitive than others.

Panel D of Table 1 shows summary statistics from the household survey data. Roughly half of the desludgings prior to privatization are mechanized, and households pay an average of 23,000 CFA (or approximately $46) per desludging. Panel E reports the average pre-privatization health outcomes from the DHS. Amongst households with at least one child under the age of 5 years, 36% in Dakar had at least one incidence of diarrhea in the two weeks prior to the survey, compared to 27% in other urban areas and 27% in other cities. Similarly, rates of cough are higher in Dakar (41 vs 31%).

### Timeline of center opening days/hours.

**Fig. 4:**
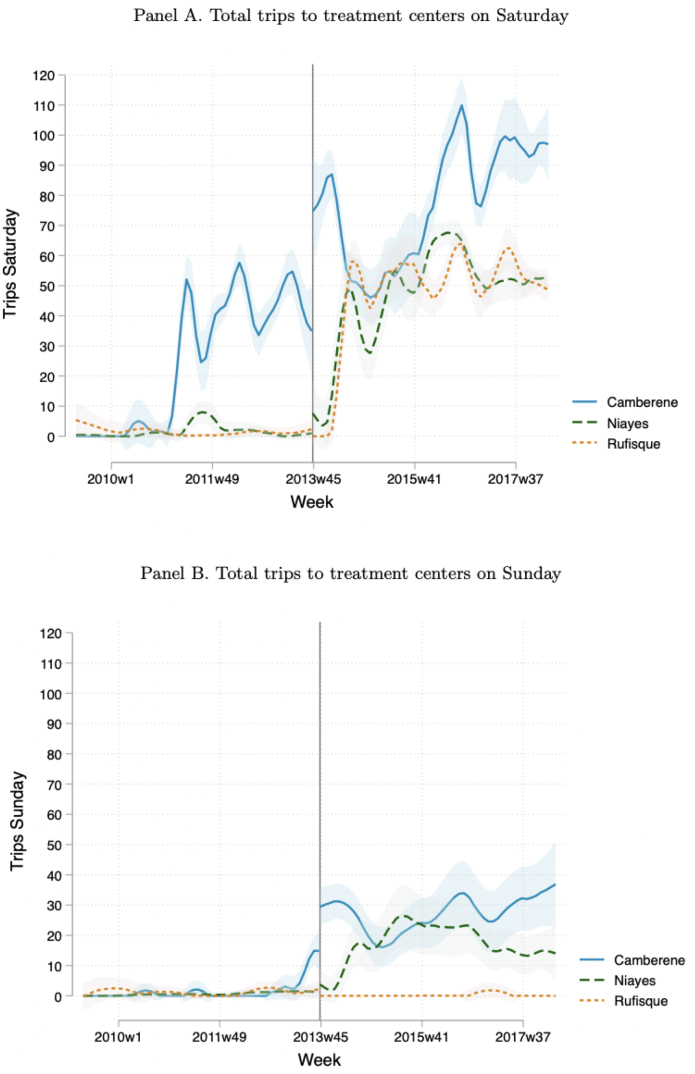
Total trips to each treatment center on Saturday and Sunday in each week. Note: Locally-weighted polynomial regressions of the total number of trips on weekend days at the week level between May 2009 and May 2018. The y-axis in panel A shows the total number of trips made to each treatment center on Saturday. The y-axis in panel B shows the total number of trips made to each treatment center on Sunday. The solid vertical line indicates the month of privatization (November 2013). The shaded area indicates 95% confidence bands.

Fig. 4 shows the total number of trips that occur in a week at each center on Saturdays (panel A) and Sundays (panel B). Prior to privatization, the center in Camberene began opening for limited hours on Saturdays in May 2011 and then expanded their opening hours on Saturdays in the weeks following privatization. The centers in Niayes and Rufisque remained closed on Saturdays prior to privatization, with the exception of a period of two months in 2011 in which the center in Niayes was open with limited hours. The centers in Camberene and Niayes also opened with limited hours on Sundays in July 2013 and April 2014, respectively. However, the center in Rufisque remained closed on Sundays following privatization.

## 4 Empirical strategy

### 4.1 Conceptual framework and predictions

Our objective is to evaluate the effect of treatment center privatization on the supply of mechanized desludgings which dump waste at those treatment centers. Treatment center operators can affect the quantity of legal desludgings performed in the market by making investments that improve the productivity of the centers, increasing the number of potential trucks that can dump each day. For instance, the operator can take actions that improve the maintenance of the center and manage its capacity in order to reduce the risk of overflowing, which can cause facility closures and congestion. They can also choose to open later in the evening and on weekends. Frequent down-times at the treatment centers have a direct effect on the number of legal mechanized desludgings that suppliers can perform. In addition to the dumping fee charged by the treatment center (which was regulated during our time period), the cost of desludging is directly proportional to the time required to dump sludge at a treatment center. As a result, truckers respond to down-times, congestion, and lack of convenient weekend and late afternoon openings by reducing their overall supply, or by dumping the sludge illegally. From a theoretical perspective, the effect of privatization on productivity is ambiguous. Before discussing our empirical strategy, it is useful to discuss the mechanisms that can lead to changes in the quantity and price of legal mechanized desludgings.

On the one hand, privatization of natural monopolies can lead to a misallocation of resources due to the presence of moral hazard and agency costs. In addition, standard models predict that privatization can lead to inefficiencies and poor performance when operators have private information about key inputs that affect the productivity of the treatment center. While the inputs of the new operators are not easily observable, the performance of the treatment centers is. We can measure performance using simple metrics such as the number of hours per day they are open, or the number of trucks that visit per day. This should in principle alleviate moral hazard concerns.

On the other hand, there exists a large economic literature showing that privatization of natural monopolies can lead to efficiency gains. Viscusi et al. (2005) argue that the reversal of the trend towards nationalization of the 1980s and 1990s led to efficiency gains in the management of assets; particularly in the energy, telecommunications, and transportation sectors. Galiani et al. (2005) show efficiency gains in the water sector in Argentina due to privatization. Staff was cut by 48% and the company went from sustaining losses to becoming profitable. The main mechanism invoked is that government-run enterprises are motivated by factors that are often unrelated to cost minimization or revenue maximization.

The delegation of the treatment centers to management by the partnership of Delta and Vicas represents a potentially large change in the operator’s objective function, relative to that of a government-run enterprise, since the new owners are also the largest suppliers of mechanized desludging in the city. As a result of privatization, the two companies became vertically integrated, controlling assets at both ends of the supply chain. A vertically-integrated operator directly benefits from improvements in the productivity of the treatment center, since it has a direct impact on the profitability of its own retail operation, and indirectly affects the profitability of its upstream operation by increasing the supply from other retail operators. In other words, since Delta and Vicas earn profits from both their downstream and upstream operations, they have a private incentive to increase the volume of sludge dumped at the treatment center. In contrast, a non-integrated utility earns profits only from the operation of the treatment center, and therefore does not internalize any effects that reducing dumping costs may have on the profitability of downstream firms. In this situation, the effect of privatization would depend crucially on the compensation of the privately-owned operator, and agency frictions could lead to inefficiencies.

A third factor determining the sign of the effect of privatization on market supply is related to the ability of the new integrated owner to limit access to rival downstream providers. In particular, we may be concerned that Delta and Vicas will engage in ‘input foreclosure.’ A vertically-integrated supplier has an incentive to favor their own trucks in order to gain a competitive advantage in the retail market. Input foreclosure can also be partial. For example, the upstream supplier (the treatment center operator) can raise the cost of accessing the treatment center to non-integrated downstream firms relative to integrated downstream firms. Dumping fee discrimination is unlikely to occur in our context, since the government retained control over the ‘sticker’ price of accessing the treatment centers. However, it is possible that Delta and Vicas trucks received special treatment post-privatization, for example by allowing drivers to pay the (cumulative) dumping fees at the end of the month (instead of per visit), or by opening a special lane for integrated suppliers. Both actions would lead to a more pronounced reduction in downstream desludging costs for trucks affiliated with Delta and Vicas.

The goal of our empirical analysis is to shed light on the relative importance of these three mechanisms. Since we do not have access to direct measures of investments by the treatment center operators, we evaluate the effect of privatization on productivity by measuring changes in the number of trips made by different operators. Since privatization did not lead to a reduction in the dumping fee, we interpret any increase in the average number of trips as a result of the reduction in congestion and down-times. To measure the overall impact of privatization, we start our analysis by measuring changes in the total number of trips to treatment centers taken by trucks. We then exploit variation across firms to test the hypothesis that privatization led to larger productivity gains for the vertically integrated suppliers.

We use different measures of company and truck supply to conduct our analysis. We first present results at the company–month level for companies active in the industry before privatization. We estimate impacts on the extensive margin including the number of trucks and the intensive margin including the productivity of those trucks (i.e., trips per truck per month). The intensive margin truck-level results are the most direct way to observe the impact of privatization on truckers’ ability to complete more jobs.

We next consider several ways in which privatization may have impacted the way that the truckers were able to perform their work: the number of trips per day that the truck works, the number of days worked per month, the number of Saturdays and Sundays that trucks work (which tend to be more convenient for clients), and the regional concentration of their jobs as measured by the number of treatment centers they visit. We briefly discuss effects on entry and exit, though our data makes it difficult to say a lot about this.

Finally, we estimate the welfare impacts of privatization on households by using household survey data from peri-urban Dakar. This allows us to estimate changes in the propensity to use mechanized desludging and household-level prices of mechanized desludgings. We also use DHS data to compare child health outcomes in Dakar to other secondary cities in Senegal prior to and after privatization.

### 4.2 Company-level regressions

We begin by estimating the impact of the privatization on companies’ productivity and available capital. Outcomes $Y_{csm}$ are measured for company c in seasonal month s (for example January) in year-by-month m (for example January 2013). We limit the sample to all companies that were present in the market prior to privatization. We estimate the following specification for all months between May 2009 and May 2018:

(1) $$Y_{csm}=\alpha +\beta_{1}PostPrivatization_{m}\left[+\beta_{2}DeltaVicas_{c}*PostPrivatization_{m}\right]\;+\;X_{m}^{\prime }\gamma +\mu_{c}+\nu_{s}+\tau_{m}+\epsilon_{csm}.$$

Outcomes at the company level include productivity (the probability of being active, total trips, and truck volume by company in each month), capital (number of trucks owned by the company), and intensity of use of capital (trips per active truck by company). At the month level, the number of trucks owned by company c is the total number of trucks that belong to that company and are between the first and last month that they appear in the data.^12^ The number of active trucks only includes the subset of these trucks that make at least one trip in month m.

The control variable of interest is $PostPrivatization_{m}$ which equals 1 in the month of privatization and all months thereafter, and equals 0 prior to privatization. In order to observe whether there are differential impacts of privatization on companies associated with Delta and Vicas, we estimate heterogeneous impacts by including the bracketed part of Eq. (1). This adds the interaction between the post-privatization indicator and whether the company’s owners also manage the treatment centers ($DeltaVicas_{c}$). This is only equal to 1 for the two companies Delta and Vicas. Month-level controls in X include rainfall and lagged rainfall since pits are more likely to fill up when rains are heavy. We also include in X an indicator for observations after the treatment center’s dumping fee changed. We include a linear monthly time trend $\tau_{m}$. Finally, we include company fixed effects $\mu_{c}$ to control for differences in average productivity across companies, and seasonal month fixed effects $\nu_{s}$ to control for differences in the weather and other seasonal influences. Standard errors use two-way clustering at the company and month levels. The sample includes all companies that were present in the data prior to privatization and each company has an observation for every month between their first and final appearance in the treatment center data. For many outcomes (except when stated otherwise), the outcome is given a value of 0 for any month in which no trucks owned by that company are observed at any treatment station.

### 4.3 Truck-level regressions

We then turn to the productivity of the trucks, limiting the sample to all trucks i that belong to companies present pre-privatization. We estimate the following specification for all months between May 2009 and May 2018:

(2) $$Y_{icsm}=\alpha +\beta_{1}PostPrivatization_{m}\left[+\beta_{2}DeltaVicas_{c}*PostPrivatization_{m}\right]\;+\;X_{m}^{\prime }\gamma +\eta_{ic}+\nu_{s}+\tau_{m}+\epsilon_{icsm}.$$

Outcome variables $Y_{icsm}$ include whether the truck does at least one job and the number of jobs that the truck does in a month in which it was active (a month in which it performed at least one job). We further consider how many different centers the truck visited that month, how many Saturdays or Sundays the truck worked that month, and the number of days the truck worked in that month. We include the same control variables as in the company-level regressions, in addition to truck fixed effects ($\eta_{ic}$). Standard errors use two-way clustering at the truck and month levels. As in the company-level regressions, we test for differential impacts of privatization based on whether Delta or Vicas owns the truck ($\beta_{2}$).

## 5 Results

### 5.1 Company-level productivity

We find that privatization had a substantial impact on the productivity of desludging companies. Column (1) of Table 2 shows that companies were 7 pp more likely to do at least one trip in a month after privatization. According to column (3), after privatization desludging companies did 51% more jobs per month, or 8 more jobs per truck per month according to column (5).^13^ Privatization has a differential impact on Delta and Vicas relative to other companies. Before privatization, Delta and Vicas were relatively more active than other companies, each conducting 115 jobs per month ($e^{4.74}$) compared to 16 jobs per month ($e^{2.76}$) for the other companies. Privatization leads Delta and Vicas to conduct 27% more jobs per month (51.8–24.6 in column (4)), about half the size of the productivity increase for other companies.

Whereas other companies are more likely to be active in a month post-privatization, Delta and Vicas were both already 100% active (doing at least one job in every month) before privatization and continue to be afterwards. Delta and Vicas also show relatively smaller increases in per-truck productivity, with a 21% increase in the number of jobs per truck per month ((8.1–3.6)/21) compared to a 45% increase for other companies (8.1/18).

**Table 2:** Monthly probability of being active, log(Trips), and trips per active truck by company.

	(1)	(2)	(3)	(4)	(5)	(6)
	P(Active)	P(Active)	Ln(Trips)	Ln(Trips)	Trips/truck	Trips/truck
Post privatization	0.0686**	0.0704**	0.510***	0.518***	7.998***	8.122***
	(0.0303)	(0.0305)	(0.117)	(0.119)	(2.572)	(2.591)
(Post privatization) × (DeltaVicas)		−0.0601***		−0.246*		−3.635*
		(0.0213)		(0.125)		(2.107)
Constant	0.896***	0.895***	2.770***	2.767***	19.88***	19.84***
	(0.0309)	(0.0310)	(0.148)	(0.149)	(2.604)	(2.617)

Observations	6573	6573	5834	5834	5834	5834
$R^{2}$	0.214	0.215	0.568	0.568	0.443	0.444
*p*-value Post priv. + Post priv. × Delvic = 0		0.74		0.05		0.12
Sample	Full	Full	Full	Full	Full	Full
Mean dep. var. before Nov 2013 (Delvic)	1.00	1.00	4.74	4.74	21.11	21.11
Mean dep. var. before Nov 2013 (Others)	0.85	0.85	2.76	2.76	18.07	18.07
Controls	X	X	X	X	X	X
Linear timetrend	X	X	X	X	X	X
Month of year FE	X	X	X	X	X	X
Company FE	X	X	X	X	X	X

Notes: OLS estimates of Eq. (1). Sample includes observations between May 2009 and May 2018 for companies that existed prior to privatization. Dependent variables are whether the company made at least one trip in that month, the log of the number of trips the company made, and the number of trips divided by the number of trucks that made trips. Observations are at the company–month level. *Post privatization* equals one for all observations after November 2013. *DeltaVicas* equals one if the company is Delta or Vicas (the two largest companies that manage the privatized centers). All specifications include a linear time trend and fixed effects for month of year (s) and company (c). Controls include rainfall, lagged rainfall, and an indicator for observations following the dumping fee increase in January 2010. We present p-values of the test that the effect of privatization on Delvic is zero (sum of the privatization and interaction coefficients). Standard errors are clustered by company and month. * Significant at 10 percent level; ** Significant at 5 percent level; *** Significant at 1 percent level.

A natural question to ask is whether privatization affected the turnover of firms in the market. It is difficult to answer this question convincingly with our data however. First, the number of firms entering and exiting is quite small for statistical analysis, and it is difficult to identify the precise entry or exit date of each company. Second, there is measurement error in the company names due to (1) changes in the way company names were recorded at the end of 2015 and (2) typos in company names during the whole study period.

Perhaps the simplest and most interesting way to address this is to point out the following numbers. During the year prior to privatization (November 2012–October 2013), we identify four entrants and five companies that exit. This is compared to an average of 8.5 entries and 5.5 exits per year in the two prior years (2010–2012). During the year after privatization, the number of new entrants was eight, but the number of exits doubled to 12 (with a total of 74 companies in the market just before privatization). Industry turnover then went back to normal in 2015.

This is suggestive evidence that privatization might have triggered the exit of some companies. That said, the overall supply of providers did not decrease. Instead, the data reveals that the number of active trucks increased by 14 from 146 to 160 following privatization, consistent with new capital investments. The years before that saw a stable number of trucks (148 and 147). Companies that exited in the year after privatization were all single-truck operators. To the extent that the size and age of the fleet is a good indicator of productivity, this is consistent with the possibility that privatization of the treatment centers induced a reallocation of business towards more efficient companies, by causing the exit of a group of small firms operating older trucks.

Table 3 suggests that most companies do not invest in new trucks following privatization. However, Delta and Vicas are significantly more likely than other companies to get new trucks, investing in 3 additional trucks after privatization. Appendix Table A6 shows regressions estimating the impact of privatization using shorter time windows. The effect of privatization on truck acquisition is not immediate. The estimated effect within nine months reflects an insignificant 10% increase for Delta and Vicas relative to other firms, which is roughly 38% of the overall estimated effect for the full sample period. The increase within two and six months is even smaller. While we would not expect privatization to have an immediate impact on capital acquisition, we cannot rule out that the total estimated relative effect of privatization on truck acquisition for Delta and Vicas was not also affected by the loan guarantee program that is discussed in Section 2.1.

**Table 3:** Number of trucks owned by company and month.

	(1)	(2)	(3)	(4)
	Trucks	Trucks	Log(Trucks)	Log(Trucks)
Post privatization	0.0110	−0.0801	−0.0143	−0.0221
	(0.105)	(0.113)	(0.0397)	(0.0402)
(Post privatization) × (DeltaVicas)		3.035***		0.260***
		(0.565)		(0.0786)
Constant	1.331***	1.365***	0.216***	0.219***
	(0.224)	(0.203)	(0.0589)	(0.0586)

Observations	6573	6573	6573	6573
$R^{2}$	0.854	0.868	0.865	0.866
*p*-value Post priv. + Post priv. × Delvic = 0		0.00		0.00
Mean dep. var. before Nov 2013 (Delvic)	6.81	6.81	1.90	1.90
Mean dep. var. before Nov 2013 (Others)	1.86	1.86	0.38	0.38
Controls	X	X	X	X
Linear timetrend	X	X	X	X
Month of year FE	X	X	X	X
Company FE	X	X	X	X

Notes: OLS estimates of Eq. (1). Sample includes observations between May 2009 and May 2018 for companies that existed prior to privatization. Dependent variables are the number of trucks owned by company *c* in month *m* and its log. Observations are at the company–month level. *Post privatization* equals one for all observations after November 2013. *DeltaVicas* equals one if the company is Delta or Vicas (the two largest companies that manage the privatized centers). All specifications include a linear time trend and fixed effects for the month of year (*s*) and company (*c*). Controls include rainfall, lagged rainfall, and an indicator for observations following the dumping fee increase in January 2010. We present p-values of the test that the effect of privatization on Delvic is zero (sum of the privatization and interaction coefficients). Standard errors are clustered by company and month. * Significant at 10 percent level; ** Significant at 5 percent level; *** Significant at 1 percent level.

### 5.2 Truck-level productivity

We investigate the impact of privatization on the probability of a truck being active in a given month and the number of trips done per truck in a month in Table 4. This provides us with an estimate of the overall increase in business that companies are able to do with one unit of capital. In columns (1) and (2), we find a positive effect of privatization on the probability of a given truck being active. This suggests that companies are maintaining trucks and keeping them on the streets more following privatization. While privatization may not convince the average company to purchase new trucks, companies do appear to keep their fleet active more of the time. On average, privatization results in each truck doing 10.1 more trips per month seen in column (3). This is an extremely large effect—a 58% increase relative to the pre-privatization mean seen in column (5). This increase in trips may be because of decreased wait times at the treatment centers, centers being open more hours and days per week, improved maintenance of the centers, or faster repairs of damaged trucks.^14^

Trucks affiliated with Delta and Vicas do not see larger increases in trips per month than trucks affiliated with other companies. In fact, the average truck affiliated with Delta or Vicas is 10% less likely to be active in a month after privatization (.116-.217 in column (2)), compared to an increase of 12% for trucks owned by other companies. Combined with the result in Table 3 showing that Delta and Vicas invested in new trucks post-privatization, this could indicate a preference among those two companies to use newer trucks, which may be more fuel efficient or reliable, as much as possible.

**Table 4:** Monthly probability of being active and log(Trips) by truck.

	(1)	(2)	(3)	(4)	(5)	(6)
	P(Active)	P(Active)	Trips	Trips	Ln(Trips)	Ln(Trips)
Post privatization	0.0941***	0.116***	10.07***	9.553***	0.580***	0.593***
	(0.0241)	(0.0255)	(2.026)	(2.117)	(0.0794)	(0.0823)
(Post privatization) × (DeltaVicas)		−0.217***		4.767		−0.119
		(0.0561)		(5.018)		(0.140)
Constant	0.916***	0.915***	23.72***	23.79***	2.718***	2.716***
	(0.0299)	(0.0297)	(2.659)	(2.625)	(0.119)	(0.119)

Observations	15308	15308	12371	12371	12371	12371
$R^{2}$	0.224	0.229	0.481	0.482	0.394	0.394
*p*-value Post priv. + Post priv. × Delvic = 0		0.06		0.00		0.00
Mean dep. var. before Nov 2013 (Delvic)	0.89	0.89	20.80	20.80	2.71	2.71
Mean dep. var. before Nov 2013 (Others)	0.76	0.76	19.24	19.24	2.41	2.41
Controls	X	X	X	X	X	X
Linear timetrend	X	X	X	X	X	X
Month of year FE	X	X	X	X	X	X
Truck FE	X	X	X	X	X	X

Notes: OLS estimates of Eq. (2). Sample includes trucks observed between May 2009 and May 2018 that belong to companies that existed prior to privatization. Dependent variables are the probability of making at least one trip, the number of trips made, and its log by truck *i* to any treatment center in month *m*. Observations are at the truck–month level. *Post privatization* equals one for all observations after November 2013. *DeltaVicas* equals one if the company is Delta or Vicas (the two largest companies that manage the privatized centers). All specifications include a linear time trend and fixed effects for month of year (*s*) and truck (*i*). Controls include rainfall, lagged rainfall, and an indicator for observations following the dumping fee increase in January 2010. We present p-values of the test that the effect of privatization on Delvic is zero (sum of the privatization and interaction coefficients). Standard errors are clustered by company and month. * Significant at 10 percent level; ** Significant at 5 percent level; *** Significant at 1 percent level.

### 5.3 Summary and mechanisms

Overall, the previous sections showed that companies and trucks were both more likely to make at least one trip to a treatment center in a month, and also, conditional on doing at least one trip, made more trips in that month. These increases in productivity are not limited to Delta and Vicas. If anything, the effects on the other smaller companies are even larger and more significant both economically and statistically. Although we find some evidence that Delta and Vicas benefited from privatization (e.g., new trucks and slightly more trips per month), we do not find that the company expanded its supply more than other suppliers. Instead, other companies on average increased their monthly output by a higher proportion than Delta and Vicas. This is consistent with important efficiency gains from vertical integration, and limited or zero foreclosure. We also find no support for theories predicting that privatization of natural monopolies causes important productivity losses. This is not to say that agency costs are not present. Instead our results imply that productivity increases caused by profit maximization and vertical integration outweigh any agency costs associated with moral hazard. For instance, it is possible that further improvements in productivity could be realized using more efficient compensation contracts, but our analysis cannot shed light on these potential gains.

Although our data does not allow us to measure investments directly, we can evaluate the effect of privatization on the fraction of days that centers are open and the hours they are open on those days. In addition to being in better repair and less likely to close down under privatization, desludgers report that after privatization treatment centers are open an additional hour per day and are more often open on Saturdays and Sundays.^15^ We test the impact of privatization on the number of Saturdays and Sundays that trucks made trips to a treatment center by month in Table 5. We find that truckers worked 0.55 more Saturdays in a month post-privatization, and 0.43 more Sundays in a month. Delvic-affiliated trucks increase their Saturday trips by less than other firms — this may be because those trucks already took more advantage of limited station availability on Saturdays, and many of their trucks operate in areas closer to the Camberene station which was more consistently open on Saturdays prior to privatization.

It should be noted that we only observe if a truck operator made a trip to a treatment center and not whether they conducted a latrine desludging. While there is the possibility that, before privatization, truck operators conducted desludgings on Saturdays or Sundays when the dumping sites were closed, interviews with truck operators suggest that the waste is relatively thick directly after a desludging and risks damaging the pump motor if it thickens over time. As a result, storing sludge in trucks overnight is not a common practice. If they did a weekend desludging, they would likely dump the sludge illegally outside a treatment center. On average, trucks dumped sludge at treatment centers an additional 3.8 days a month following privatization than they had prior to privatization, which is an increase of 55%. The increase in the number of days worked is due both to the increases in weekend openings (0.55+0.43 extra weekend days a month), as well as fewer days that trucks are left idle due to lack of work or maintenance.

Prior to privatization, desludgers often cited concerns about treatment centers going offline due to repairs, so they were less willing to serve regions of the city that were farther from their main treatment center. As trucks are parked at a garage that is shared with other truckers who typically use the same treatment center, the drivers receive information quickly on closures at their preferred treatment center, but may be surprised by closures at treatment centers that are farther away. Because gas is a major downstream input cost, the risk of arriving at a closed center might make them less likely to expand their territory to neighborhoods which are more proximate to their non-preferred treatment centers.

**Table 5:** Worked Saturdays, Sundays, and number of days, and centers visited by truck.

	(1)	(2)	(3)	(4)	(5)	(6)	(7)	(8)
	Work Sat	Work Sat	Work Sun	Work Sun	Days worked	Days worked	N centers	N centers
Post privatization	0.549***	0.597***	0.432***	0.441***	3.840***	4.162***	0.188***	0.181***
	(0.117)	(0.119)	(0.0749)	(0.0768)	(0.565)	(0.587)	(0.0529)	(0.0568)
(Post privatization) × (DeltaVicas)		−0.465**		−0.0834		−3.148***		0.0545
		(0.187)		(0.141)		(1.197)		(0.0893)
Constant	0.215*	0.211*	0.0324	0.0317	9.057***	9.032***	1.595***	1.596***
	(0.111)	(0.112)	(0.0688)	(0.0691)	(0.812)	(0.820)	(0.115)	(0.115)

Observations	15308	15308	15308	15308	15308	15308	11226	11226
$R^{2}$	0.416	0.418	0.387	0.387	0.417	0.419	0.424	0.424
*p*-value Post priv. + Post priv. × Delvic = 0		0.51		0.02		0.39		0.00
Mean dep. var. before Nov 2013 (Delvic)	0.71	0.71	0.05	0.05	8.71	8.71	1.97	1.97
Mean dep. var. before Nov 2013 (Others)	0.48	0.48	0.05	0.05	7.02	7.02	1.47	1.47
Controls	X	X	X	X	X	X	X	X
Linear timetrend	X	X	X	X	X	X	X	X
Month of year FE	X	X	X	X	X	X	X	X
Truck FE	X	X	X	X	X	X	X	X

Notes: OLS estimates of Eq. (2). Sample includes trucks observed between May 2009 and May 2018 that belong to companies that existed prior to privatization. Dependent variables are the number of Saturdays truck *i* made a trip to any treatment center in month *m*, the number of Sundays truck *i* made a trip to any treatment center in month *m*, the number of days truck *i* made at least one trip to a treatment center in month *m*, and the number of treatment centers that truck *i* visited in month *m* (the sample for that outcome is limited to trucks that made at least three trips in the month). Observations are at the truck-month level. *Post privatization* equals one for all observations after November 2013. *DeltaVicas* equals one if the company is Delta or Vicas (the two largest companies that manage the privatized centers). All specifications include a linear time trend and fixed effects for the month of year (*s*) and truck (*i*). Controls include rainfall, lagged rainfall, and an indicator for observations following the dumping fee increase in January 2010. We present p-values of the test that the effect of privatization on Delvic is zero (sum of the privatization and interaction coefficients). Standard errors are clustered by company and month. * Significant at 10 percent level; ** Significant at 5 percent level; *** Significant at 1 percent level.

We test whether desludgers expand their territory following privatization by estimating the effect of privatization on the number of treatment centers that trucks visit in a month. Table 5 provides suggestive evidence that following privatization truckers may less consistently work in their own territories. After privatization, trucks increase the number of centers that they visit in a month by 0.19, which represents a 12% increase. This suggests a decrease in the distance cost associated with the supply of downstream desludging services, and a potential increase in the competitiveness of the market through a reduction in spatial differentiation. The increase in competitiveness is borne out through substantial downstream price decreases, as shown in the household regressions below in Table 6. The impact on the number of centers visited is larger for Delta and Vicas—suggesting that they also expand their territory, though the coefficient on the interaction term is insignificant.

## 6 Welfare impacts

We evaluate the extent to which the increased quantity of sludge dumped at treatment centers following privatization was also reflected in a larger reported market share for mechanized desludging among households. We also evaluate whether the potential increase in upstream supply and cost savings were reflected in lower downstream prices for households purchasing mechanized desludgings. Finally, the main welfare benefit of an increase in dumping at treatment centers, whether due to an increase in mechanized desludgings or a decrease in illegal unsanitary dumping, is a more sanitary environment. To the extent that sanitation improves, diarrhea rates may also go down. We compare changes in children’s diarrhea prevalence reported in Dakar and in secondary cities in Senegal.^16^

### 6.1 Household utilization and prices

As the ultimate goal of the privatization policy was to improve sanitation in Dakar, we investigate its impacts on the prices that consumers pay for mechanized desludging and the share of mechanized versus manual desludgings that they purchase. We use household surveys conducted before and after privatization to evaluate the impact of privatization on these outcomes. Specifically, we conducted four household surveys in Dakar in Sept-Dec 2012, Feb-May 2014, Jun-Jul 2014, and Mar-Jun 2015. In each survey, we have retrospective data on the households’ most recent desludging including the type (mechanized or manual), price the household paid, and the month of the desludging. To reduce recall bias, we consider households’ most recent desludging that took place within 12 months of the survey date. In total, we have a sample of 9,033 households across the four surveys, of which 85% purchased at least one desludging in the 12 months prior. As a result, we observe 7,684 desludgings between October 2011 and May 2015. To estimate the impact of privatization on household-level desludging prices and demand for mechanized desludging, we estimate the following specification:

(3) $$y_{izsm}=\alpha +\beta_{1}PostPrivatization_{m}+\upsilon_{s}+\phi_{z}+\tau_{m}+\epsilon_{izsm}$$

where $y_{izsm}$ is an indicator for whether household *i* in subzone z purchases a mechanized desludging conditional on purchasing any desludging or the price that they paid for the mechanized desludging in month m. We include month of year and subzone fixed effects ($\upsilon_{s}$ and $\phi_{z}$), a linear monthly time trend $\tau_{m}$, and cluster standard errors at the subzone and month level using two-way clustering. Because we do not have reliable data on which company the household used in the cases when they purchased a mechanized desludging, we do not include a control for *DeltaVicas* or its interaction.

We test the impact of privatization on the share of desludgings done that were mechanized in Table 6. Privatization increased the share of mechanized desludgings in the neighborhoods we surveyed by 2.4 pp (4.6%) relative to all available desludgings (Column (1)). The price households paid for a mechanized desludging went down by five percent. This suggests that households switched from manual desludgings to mechanized desludging due to increased supply and lower prices. Given the relatively small increase in demand, this is suggestive evidence that the large increase in sludge dumped at the treatment centers was primarily caused by a decrease in illegal dumping by the trucks, alongside a decrease in manual desludgings by households.

To the extent that the market is fully competitive, we should expect full pass-through of changes in input costs to the consumer.^17^ Truckers have lower costs if they experience shorter lines at treatment centers, pay fewer bribes due to dumping illegally less often, and drive less far to do illegal dumping. If there is market power, changes in input costs will not be fully passed through. Therefore, our estimate of the impact of privatization on the price downstream households pay for a mechanized desludging provides a lower bound for the impact of privatization on desludger input costs. Table 6 shows that the average price of a mechanized desludging as reported by households went down by 1071 CFA following privatization. This effect is approximately 5% of the mean price of a mechanized desludging prior to privatization.

**Table 6:** Share mechanized and mechanized prices reported by households.

	(1)	(2)	(3)
	P(Mech)	Price	Ln(Price)
Post privatization	0.0237***	−1071.0*	−0.0533**
	(0.00625)	(523.6)	(0.0238)

Observations	7684	3840	3840
$R^{2}$	0.149	0.224	0.231
Mean dep. var.	0.52	23357.52	10.00
Rainfall controls	X	X	X
Linear timetrend	X	X	X
Month of year FE	X	X	X
Subzone FE	X	X	X
Survey FE	X	X	X
Sample	Last	Last mech.	Last mech.
	desludging	desludging	desludging

Notes: OLS estimates of Eq. (3). Sample includes desludgings reported in the household survey data in the year before the survey between October 2011 and May 2015. Dependent variables are whether the household chose mechanized desludging for that desludging and the price (and log price) paid for the mechanized desludging for household *i* in subzone *z* in month *m*. The price is winsorized at 10,000 and 50,000. Observations are at the household-desludging-month level. *Post privatization* equals one for all observations after November 2013. All specifications include a linear time trend and fixed effects for the subzone, survey round, and month of year of the desludging. Controls include rainfall and lagged rainfall. Standard errors are clustered by subzone and month. * Significant at 10 percent level; ** Significant at 5 percent level; *** Significant at 1 percent level.

### 6.2 Health outcomes

Improved sanitation, either due to an increase in mechanized desludgings or a decrease in illegal unsanitary dumping, may reduce negative health externalities. We analyze the impact of privatization on the incidence of diarrhea and use the incidence of cough as a placebo test. Improved health can lead to increases in human capital and sustained economic growth.

To estimate the extent to which privatization improved children’s health, we estimate panel regressions comparing diarrhea rates in Dakar to those in secondary cities in Senegal. The outcome variable is an indicator variable for any under 5 child in household *i* experiencing an episode of diarrhea in the two weeks prior to the survey in seasonal month *s* and year *t* in urban region *r*. Our regressions take the form:

(4) $$Illness_{irst}=\overset{2019}{\underset{t=2010}{\sum }}\beta_{t}\cdot Dakar_{i}+\Gamma X_{irst}+\gamma_{t}+\lambda_{s}+\nu_{r}+\epsilon_{irst}.$$

The main control of interest, $Dakar_{i}$, is an indicator variable that equals 1 if the household lives in Dakar, and 0 otherwise. We further control for time-varying household characteristics including the number of children under 5, the household’s water source, toilet type, if the toilet is shared with other families, the age and education of the household head, and an index of household wealth. Region fixed effects control for underlying differences in the incidence of illness in each region.^18^ The year fixed effects absorb changes in illness incidence that affect all regions, and month fixed effects account for seasonal variation in diarrhea risk. The main coefficients of interest are $\beta_{t}$, which measure the differential change in illness incidence in Dakar (where privatization occurred) in year *t* relative to households in other urban areas in Senegal. In addition, we identify urban clusters that are located in cities that have a similar population and density to Dakar and estimate Eq. (4) on this subsample. As a falsification test, we also estimate this regression for an alternative outcome (cough incidence), which should be less affected by improved sanitation.

Appendix Figure A1 presents trends in child diarrhea and cough incidence in Dakar and the other cities in Senegal. Panels A and B demonstrate that the trend in diarrhea and cough incidence is indeed similar in Dakar relative to other cities in Senegal prior to privatization, including when we control for household conditions that may vary between Dakar and other urban areas. This provides further evidence that the selected cities serve as a suitable control group to Dakar.^19^

**Fig. 5:**
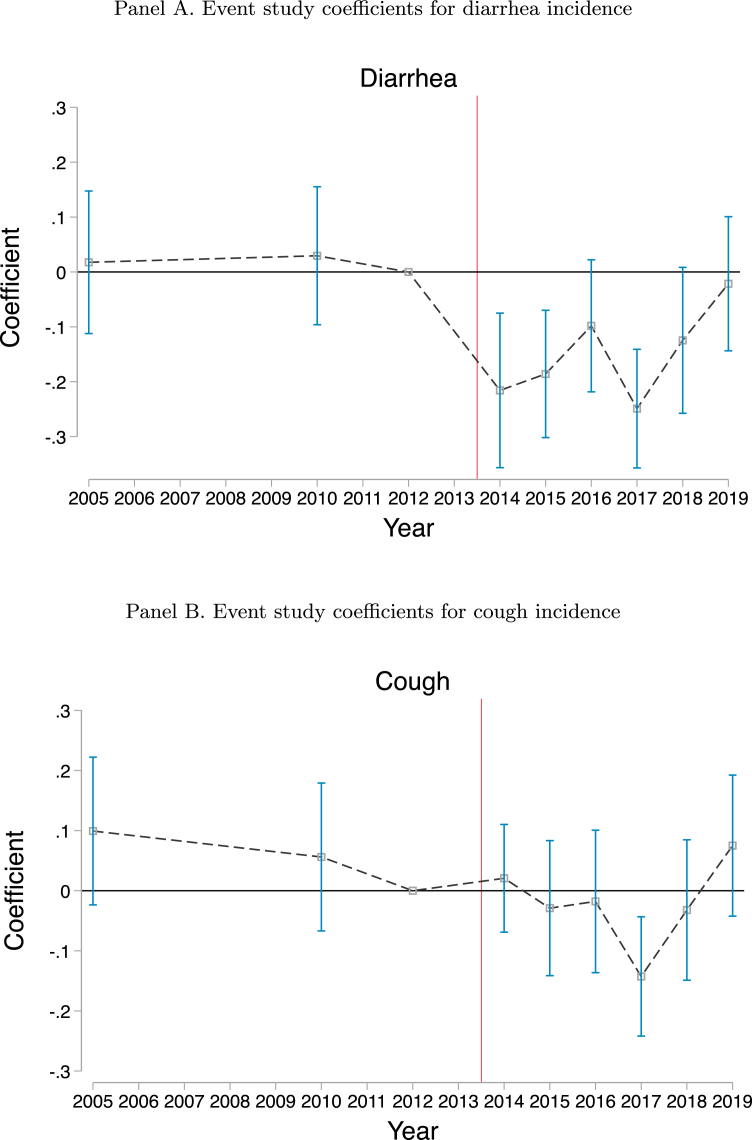
Illness incidence amongst children under 5 in Dakar vs other urban areas in Senegal. Note: Coefficients from Eq. (4). Dependent variables are the incidence of diarrhea (Panel A) and the incidence of cough (Panel B). The omitted category is 2012, the year leading up to the 2013 privatization (indicated with a vertical line). The bars indicate 95% confidence intervals based on standard errors clustered by survey cluster.

Fig. 5 plots the estimated coefficients of Eq. (4) for both diarrhea (Panel A) and cough (Panel B).^20^ The figure reveals that diarrhea incidence declines in Dakar following privatization relative to other urban areas in Senegal, and remains persistently lower thereafter. Privatization is associated with a 22 percentage point decline in diarrhea incidence relative to other urban areas in Senegal. In contrast, we find little effect of privatization on the incidence of cough.

## 7 Conclusion

Effective oversight of public goods is difficult, and poor government management can lead to negative impacts on downstream sectors. Maintenance issues, wait times, and unpredictability can impact productivity, leading to higher input costs and higher downstream prices. In sectors such as sanitation in which there are substantial health externalities from lack of access, the welfare effects of poor management can be significant.

We show that Senegal’s privatization of the management of its sewage treatment plants led to a substantial increase in the amount of sewage dumped at legal treatment centers. Our data does not allow us to clearly decompose this effect between the increase in the use of mechanized desludging and the decrease in illegal dumping by trucks. However, both practices result in similar poor disposal of sewage and result in negative health impacts in urban communities.

We show suggestive evidence of an impact from privatization on water-borne diseases. While there are other policies and unobservable factors that may have simultaneously reduced diarrhea in Dakar relative to other regions, the immediate and persistent effect in the years directly following privatization suggest that the large increase in sanitary waste disposal likely contributed to improved health outcomes in the city. The impact of privatization on welfare is therefore quite large.

Downstream small businesses benefit from improved efficiency of key input factors. We show that the average downstream trucking company did more jobs per month. This effect is caused both by the companies’ ability to do more jobs per day, and because companies use their capital more intensively—trucks are actively engaged in the market a larger proportion of months in the year and trucks work more of the month.

## CRediT authorship contribution statement

**Joshua W. Deutschmann:** Conceptualization, Data curation, Formal analysis, Investigation, Methodology, Software, Visualization, Writing. **Jared Gars:** Conceptualization, Data curation, Formal analysis, Methodology, Software, Validation, Visualization, Writing. **Jean-François Houde:** Conceptualization, Formal analysis, Funding acquisition, Methodology, Project administration, Supervision, Writing. **Molly Lipscomb:** Conceptualization, Data curation, Formal analysis, Funding acquisition, Methodology, Project administration, Supervision, Validation, Writing. **Laura Schechter:** Conceptualization, Data curation, Formal analysis, Funding acquisition, Methodology, Project administration, Supervision, Writing.

## Data Availability

Replication data are available at the UVA Dataverse: https://doi.org/10.18130/V3/XL3CUA.

## Appendix A Supplementary data

MMC S1 .

## Footnotes

1 The sewage treatment centers were operating far above their designed maximum daily capacity, and maintaining that operation required active management of infrastructure (Sene, 2017).

2 Part of the low baseline activity may be due to measurement error and trucks doing non-desludging related work such as flood control, but we find large gains in the number of active trucks at the company–month level following privatization, demonstrating that prior to privatization there was substantial excess capacity that was only harnessed once treatment centers were privatized.

3 Manual waste disposal is illegal in many countries, including Senegal, and has strong negative effects on the households engaging in it as well as on their neighbors (Deutschmann et al., 2022, Gertler et al., 2015, Johnson and Lipscomb, 2021).

4 Higher incidence of diarrhea may lead to higher incidence of respiratory disease, but the primary impact of poor sanitation is through diarrhea (Mara et al., 2010). Any impact of sanitation on respiratory disease would make the difference in effects between diarrhea and respiratory disease a lower bound on the total impact.

5 These are the only legal dumping sites available in Dakar for the majority of the period we study. A fourth center, at Dakar’s far north-east edge in Tivaouane Peulh, opened in October 2017.

6 The centers, designed to process about 240 m${}^{3}$ of sludge per day, were collecting more than four times that amount in 2012 (Sene, 2017).

7 The public record of the call for proposal launch can be found here: http://www.marchespublics.sn/index.php?option=com_plan&task=viewrealisationentame&idtype=&idautorite=108&year=2012&tagproc=1835163&ref=C_DG_044&Itemid=105. Of eight initially interested firms, only the Delvic partnership managed to submit a complete bid for the contract: https://www.onasbv.sn/app/uploads/2014/07/Rapport-Avancement-Num-2.pdf.

8 This decline in 2010 might be an expected result of the increase in the number of active trucks at the same time if demand for mechanized desludging services is inelastic. In other work, we show that the elasticity of demand for mechanized desludging services in Dakar is −2.2 (Deutschmann et al., 2022), which suggests that demand is quite elastic and so that explanation may be less reasonable.

9 From May to November 2017, ONAS ran a city-wide subsidy program offering households discounts on mechanized desludgings. There were 5350 subsidized desludgings (Deutschmann, 2022), which represents 14% of all recorded trips to the treatment centers during that time period. The effects of privatization we observe are not concentrated in these months.

10 We constructed subzones by dividing each of the 10 arrondissements in Dakar into five roughly equally sized blocks.

11 We have also considered a *Density* definition according to which clusters must have a population density that is at least as large as the lowest population density sampled point in Dakar (www.worldpop.org, 2022, OECD/SWAC, 2020). In practice, the *Cities* definition and the *Density* definition result in a nearly identical sample.

12 To reduce errors associated with the misrecording of license plates, we exclude the first and last ten times that a license plate appears in the data.

13 Results looking at volume in meters cubed rather than in the number of trips can be found in Appendix Table A5.

14 Results looking at volume in meters cubed rather than in the number of trips can be found in Appendix Table A7 .

15 There were some weekend day openings prior to privatization, but those were infrequent and inconsistent.

16 Other papers looking at the health effects of privatization of water and sewerage find that the poor benefit the most (Galiani et al., 2005, Kosec, 2014), seemingly due to an increase in connections among these populations. Our setting does not involve connections, but instead truckers dumping in treatment centers rather than in neighborhoods, so we do not expect to find heterogeneous impacts.

17 In other work, we find that the market is not fully competitive (Houde et al., 2021). To the extent that the market is less than fully competitive, price changes for consumers represent a lower bound on cost changes to suppliers.

18 We use region fixed effects instead of strata fixed effects because our sample is limited to urban regions. The DHS data in Senegal followed a stratified sampling method based on the classification of households as urban vs rural as well as by region. Our data includes the city of Dakar (as treatment) and urban clusters that act as controls. We also include an analysis that limits the control clusters to the seven largest cities in Senegal: Diourbel, Kaolack, Louga, Saint-Louis, Tambacounda, Thies, and Ziguinchor. These cities have limited sewerage infrastructure and most have no functioning treatment center for sanitary sludge disposal during the period we study.

19 Diarrhea incidence in Dakar begins significantly higher than incidence in other cities. Henderson and Turner (2020) suggest that diarrhea rates rise with density (after controlling for other factors) despite the fact that safe water and improved sanitation both improve with density. One interpretation provided by Henderson and Turner (2020) is that as density rises, the increased access to safe water and improved sanitation is not enough to offset the effect of increased crowding on contamination of food and water.

20 Appendix Table A8 presents the results for an event study regression only comparing before and after in Dakar. Appendix Table A9 reports the corresponding difference-in-differences estimates, with and without controls, for both the full urban sample and the subsample that includes only cities with a sufficiently high population density.
