# Supplementary material for: Privatization of public goods: Evidence from the sanitation sector in Senegal
Source: J Dev Econ. 2023 Jan;160:102971. doi: 10.1016/j.jdeveco.2022.102971 (PMC9756007; doi:10.1016/j.jdeveco.2022.102971)
Supplement: MMC S1 — . [file mmc1.pdf]

Appendix Tables

Table A1: Trucker reports of changes after privatization

Variable	(1) Obs	(2) Mean
Panel A: Positive changes at center		
Positive changes=1	203	0.77
Longer hours	157	0.79
Fewer days closed	157	0.71
Shorter wait line for dumping	157	0.19
Improvements to center	157	0.12
Quicker payment	157	0.06
Panel B: Negative changes at center		
Negative changes=1	203	0.24
Increased dumping costs	48	0.31
Longer wait line for dumping	48	0.27
Stricter rules about contents dumped	48	0.21
More days closed	48	0.15
Favoritism	48	0.06
Longer payment	48	0.02
Panel C: Adjustments to operating hours		
Change way work=1	204	0.80
Finish days later	163	0.81
Give more weekend appointments	163	0.75
Give more afternoon appointments	163	0.45
Accept clients from the call center	163	0.01

Note: Summary statistics from the survey with truck owners and operators in January and February 2015. Summary statistics are presented based on whether they noted positive changes (Panel A) or negative changes (Panel B), and how they adjusted their behavior as a result of longer operating hours (Panel C).

Table A2: Aggregate trips per month

	(1) Trips	(2) Ln(Trips)	(3) Ln(Trips)	(4) Ln(Trips)	(5) Ln(Trips)
Post privatization	1643.5***	0.552***	0.516***	0.541***	0.467***
	(207.3)	(0.0525)	(0.0486)	(0.0504)	(0.0412)
Constant	2316.2***	7.747***	7.553***	7.551***	7.535***
	(168.8)	(0.0589)	(0.0804)	(0.0817)	(0.0836)
Observations	109	109	63	60	57
R^2	0.908	0.919	0.880	0.847	0.818
Sample	Full	Full	9 months	6 months	2 months
Mean dep. var. pre-priv. (logs)	7.69	7.69	7.69	7.69	7.69
Mean dep. var. pre-priv (levels)	2224.26	2224.26	2224.26	2224.26	2224.26
Controls	X	X	X	X	X
Linear timetrend	X	X	X	X	X
Month of year FE	X	X	X	X	X

Note: OLS estimates of monthly aggregate trips to any treatment center. Sample in columns (1)-(2) includes all months between May 2009 and May 2018. Samples in columns (3)-(5) limited to all months between May 2009 and the 9 months, 6 months, and 2 months after privatization respectively. Dependent variables are the number of trips and the log of the number of trips made by any truck to a treatment center in month m. Observations are at the month level. *Post privatization* equals one for all observations after November 2013. All specifications include a linear time trend and fixed effects for month of year s. Controls include rainfall, lagged rainfall, and an indicator for observations following the dumping fee increase in January 2010. Robust standard errors in parentheses. * Significant at 10 percent level; ** Significant at 5 percent level; *** Significant at 1 percent level.

Table A3: Aggregate trips per month by station

	(1) Ln(Trips)	(2) Ln(Trips)	(3) Ln(Trips)	(4) Ln(Trips)	(5) Ln(Trips)	(6) Ln(Trips)
Post privatization	0.422***	0.400***	0.807***	0.767***	0.589***	0.501***
	(0.0772)	(0.0839)	(0.0936)	(0.0742)	(0.0627)	(0.0452)
Constant	6.699***	6.438***	5.341***	4.823***	7.207***	5.937***
	(0.117)	(0.262)	(0.224)	(0.314)	(0.0793)	(0.191)
Observations	108	108	98	98	108	108
R^2	0.800	0.803	0.885	0.911	0.758	0.863
Station	Camberene	Camberene	Niayes	Niayes	Rufisque	Rufisque
Mean dep. var. before Nov 2013	7.183	7.183	6.673	6.673	7.048	7.048
Controls	X	X	X	X	X	X
Linear timetrend	X	X	X	X	X	X
Month of year FE	X	X	X	X	X	X
Days open		X		X		X

Note: OLS estimates of monthly aggregate trips separately for the three treatment centers. Sample includes all months between May 2009 and May 2018. Dependent variable is the log of the number of trips made by any truck to that treatment center in month m. Observations are at the month level. *Post privatization* equals one for all observations after November 2013. All specifications include a linear time trend and fixed effects for month of year s. Controls include rainfall, lagged rainfall, and an indicator for observations following the dumping fee increase in January 2010. Robust standard errors in parentheses. * Significant at 10 percent level; ** Significant at 5 percent level; *** Significant at 1 percent level.

Table A4: Total number of households and clusters by DHS survey round (2005-2019)

	(1)	(2)	(3)	(4)	(5)	(6)
	**Dakar**		**Urban**		**Cities**	
Round	Households	Clusters	Households	Clusters	Households	Clusters
2005	357	41	1378	117	974	55
2010	334	30	1433	117	812	46
2012	153	16	799	63	452	25
2014	132	14	855	64	496	26
2015	145	16	833	68	552	28
2016	155	16	812	68	532	26
2017	409	42	1752	144	909	56
2018	172	16	831	66	595	28
2019	146	16	780	68	440	26
Total	2003		9473		5762	

Notes: Number of households and clusters in each round of the Demographic and Health Survey (DHS) between 2005 and 2019. Sample is limited to households with at least one child under 5. Columns (1)-(2) include households and clusters in urban areas in the region of Dakar. Columns (3)-(4) include households and clusters in urban areas outside of the region of Dakar. Columns (5)-(6) include households and clusters in urban areas that are located in large cities outside of the region of Dakar.

Table A5: Monthly probability of being active and volume by company

	(1)	(2)	(3)	(4)	(5)	(6)
	P(Active)	P(Active)	Ln(Volume)	Ln(Volume)	Volume/truck	Volume/truck
Post privatization	0.0686**	0.0704**	0.482***	0.492***	72.18***	73.07***
	(0.0303)	(0.0305)	(0.118)	(0.119)	(22.50)	(22.77)
(Post privatization) x (DeltaVicas)		-0.0601***		-0.286**		-26.31
		(0.0213)		(0.135)		(17.59)
Constant	0.896***	0.895***	4.704***	4.700***	113.0***	112.7***
	(0.0309)	(0.0310)	(0.135)	(0.135)	(18.75)	(18.83)
Observations	6573	6573	5834	5834	5834	5834
R^2	0.214	0.215	0.588	0.588	0.438	0.438
p-value Post priv. + Post priv. x Delvic = 0		0.74		0.15		0.03
Sample	Full	Full	Full	Full	Full	Full
Mean dep. var. before Nov 2013 (Delvic)	1.00	1.00	7.04	7.04	211.43	211.43
Mean dep. var. before Nov 2013 (Others)	0.85	0.85	4.86	4.86	144.65	144.65
Controls	X	X	X	X	X	X
Linear timetrend	X	X	X	X	X	X
Month of year FE	X	X	X	X	X	X
Company FE	X	X	X	X	X	X

Notes: OLS estimates of equation (1). Sample includes observations between May 2009 and May 2018 for companies that existed prior to privatization. Dependent variables are the probability of making at least one trip, the log of the volume dumped, and volume dumped per active truck by company c in month m. Observations are at the company-month level. *Post privatization* equals one for all observations after November 2013. *DeltaVicas* equals one if the company is Delta or Vicas (the two largest companies that manage the privatized centers). All specifications include a linear time trend and fixed effects for the month of year (s) and company (c). Controls include rainfall, lagged rainfall, and an indicator for observations following the dumping fee increase in January 2010. We present p-values of the test that the effect of privatization on Delvic is zero (sum of the privatization and interaction coefficients). Standard errors are clustered by company and month. * Significant at 10 percent level; ** Significant at 5 percent level; *** Significant at 1 percent level.

Table A6: Number of trucks owned by company and month

	(1) Log(Trucks)	(2) Log(Trucks)	(3) Log(Trucks)	(4) Log(Trucks)
Post privatization	-0.0221	-0.0330	-0.0402	-0.0536*
	(0.0402)	(0.0329)	(0.0302)	(0.0274)
(Post privatization) x (DeltaVicas)	0.260***	0.0979	0.0386	0.0133
	(0.0786)	(0.0683)	(0.0963)	(0.0825)
Constant	0.219***	0.157**	0.155**	0.153**
	(0.0586)	(0.0596)	(0.0582)	(0.0574)
Observations	6573	4108	3910	3700
R^2	0.866	0.900	0.903	0.906
p-value Post priv. + Post priv. x Delvic = 0	0.00	0.35	0.99	0.64
Mean dep. var. before Nov 2013 (Delvic)	1.90	1.90	1.90	1.90
Mean dep. var. before Nov 2013 (Others)	0.38	0.38	0.38	0.38
Controls	X	X	X	X
Linear timetrend	X	X	X	X
Month of year FE	X	X	X	X
Company FE	X	X	X	X
Sample	Full	9 months	6 months	2 months

Note: OLS estimates of equation (1). Sample in columns (1)-(2) includes observations between May 2009 and May 2018 for companies that existed prior to privatization. Samples in columns (2)-(4) limit the sample to all months between May 2009 and the 9 months, 6 months, and 2 months after privatization respectively. Dependent variable is the log of the number of trucks owned by company i in month m. Observations are at the company-month level. *Post privatization* equals one for all observations after November 2013. *DeltaVicas* equals one if the company is Delta or Vicas (the two largest companies that manage the privatized centers). All specifications include a linear time trend and fixed effects for the month of year (s) and company (c). Controls include rainfall, lagged rainfall, and an indicator for observations following the dumping fee increase in January 2010. We present p-values of the test that the effect of privatization on Delvic is zero (sum of the privatization and interaction coefficients). Standard errors are clustered by company and month. * Significant at 10 percent level; ** Significant at 5 percent level; *** Significant at 1 percent level.

Table A7: Monthly probability of being active and volume by truck

	(1) P(Active)	(2) P(Active)	(3) Volume	(4) Volume	(5) Ln(Volume)	(6) Ln(Volume)
Post privatization	0.0941***	0.116***	90.06***	90.46***	0.561***	0.577***
	(0.0241)	(0.0255)	(15.54)	(16.55)	(0.0783)	(0.0812)
(Post privatization) x (DeltaVicas)		-0.217***		-3.929		-0.150
		(0.0561)		(45.11)		(0.136)
Constant	0.916***	0.915***	116.1***	116.1***	4.608***	4.606***
	(0.0299)	(0.0297)	(18.66)	(18.64)	(0.123)	(0.123)
Observations	15308	15308	15308	15308	12371	12371
R^2	0.224	0.229	0.428	0.428	0.412	0.412
p-value Post priv. + Post priv. x Delvic = 0		0.06		0.04		0.00
Mean dep. var. before Nov 2013 (Delvic)	0.89	0.89	185.20	185.20	4.98	4.98
Mean dep. var. before Nov 2013 (Others)	0.76	0.76	115.84	115.84	4.52	4.52
Controls	X	X	X	X	X	X
Linear timetrend	X	X	X	X	X	X
Month of year FE	X	X	X	X	X	X
Truck FE	X	X	X	X	X	X

Notes: OLS estimates of equation (2). Sample includes trucks observed between May 2009 and May 2018 that belong to companies that existed prior to privatization. Dependent variables are the probability of making at least one trip, the total volume dumped, and its log by truck i in month m. Observations are at the truck-month level. *Post privatization* equals one for all observations after November 2013. *DeltaVicas* equals one if the company is Delta or Vicas (the two largest companies that manage the privatized centers). All specifications include fixed effects for the month of year (s) and truck (i). Controls include rainfall, lagged rainfall, and an indicator for observations following the dumping fee increase in January 2010. We present p-values of the test that the effect of privatization on Delvic is zero (sum of the privatization and interaction coefficients). Standard errors are clustered by company and month. * Significant at 10 percent level; ** Significant at 5 percent level; *** Significant at 1 percent level.

Table A8: Diarrhea incidence amongst children under 5 in Dakar

	(1) Diarrhea	(2) Diarrhea
Post privatization	-0.126**	-0.149**
	(0.0637)	(0.0637)
Constant	0.383***	0.0994
	(0.0497)	(0.0627)
Observations	2003	2003
R^2	0.046	0.144
Mean dep. var. before 2014	0.35	0.35
Month FE	X	X
Controls		X

Note: OLS estimates of diarrhea incidence amongst households with children under 5 years of age. Sample includes all households in Dakar with at least one child under 5. Data includes 9 waves of Senegal's DHS data (2005, 2010, 2012, 2014, 2015, 2016, 2017, 2018, and 2019). *Post privatization* equals one for all observations after the 2012 wave. All specifications include a linear time trend and month of year s. Controls include the number of children under 5, the household's water source, toilet type, if the toilet is shared with other families, the age and education of the household head, and an index of household wealth. Standard errors are clustered at the cluster (census district) level. * Significant at 10 percent level; ** Significant at 5 percent level; *** Significant at 1 percent level.

Table A9: Illness incidence amongst children under 5 in Dakar vs other urban areas in Senegal

	(1) Diarrhea	(2) Diarrhea	(3) Diarrhea	(4) Cough	(5) Cough	(6) Cough
Dakar x 2005	0.00547	0.0176	0.0436	0.0990	0.0993	0.119
	(0.0406)	(0.0392)	(0.0550)	(0.0661)	(0.0671)	(0.0812)
Dakar x 2010	0.0493	0.0295	-0.0181	0.0700	0.0561	0.0782
	(0.0295)	(0.0326)	(0.0354)	(0.0620)	(0.0635)	(0.0682)
Dakar x 2014	-0.158***	-0.216***	-0.236***	0.0575	0.0207	0.0577
	(0.0271)	(0.0299)	(0.0270)	(0.0565)	(0.0565)	(0.0578)
Dakar x 2015	-0.175***	-0.186***	-0.175***	-0.0374	-0.0290	-0.00479
	(0.0212)	(0.0195)	(0.0224)	(0.0611)	(0.0637)	(0.0654)
Dakar x 2016	-0.0680***	-0.0981***	-0.114***	-0.00234	-0.0178	0.0388
	(0.0159)	(0.0173)	(0.0194)	(0.0602)	(0.0649)	(0.0667)
Dakar x 2017	-0.218***	-0.249***	-0.239***	-0.136**	-0.143**	-0.0929
	(0.0360)	(0.0413)	(0.0422)	(0.0542)	(0.0558)	(0.0592)
Dakar x 2018	-0.111***	-0.125***	-0.142***	-0.0234	-0.0321	-0.0328
	(0.0186)	(0.0146)	(0.0267)	(0.0613)	(0.0624)	(0.0666)
Dakar x 2019	-0.00103	-0.0215	-0.0340	0.0889	0.0751	0.134*
	(0.0357)	(0.0347)	(0.0470)	(0.0720)	(0.0735)	(0.0797)
Observations	11476	11476	8160	11476	11476	8160
R^2	0.031	0.102	0.102	0.070	0.104	0.102
Mean dep. var. before 2014	0.28	0.28	0.29	0.32	0.32	0.35
Strata FE	X	X	X	X	X	X
Year FE	X	X	X	X	X	X
Month FE	X	X	X	X	X	X
Controls		X	X		X	X
Cities			X			X

Note: OLS estimates of illness incidence amongst households with children under 5 years of age. Sample includes all households with at least one child under 5. Sample in columns (1)-(2) and (4)-(5) include all households classified as urban by the DHS. Sample in columns (3) and (6) limits the sample to clusters located within large cities in Senegal. Data includes 9 waves of Senegal's DHS data (2005, 2010, 2012, 2014, 2015, 2016, 2017, 2018, and 2019). *Post privatization* equals one for all observations after the 2012 wave. All specifications include fixed effects for strata (region) r, year t, and month of year s. Controls include the number of children under 5, the household's water source, toilet type, if the toilet is shared with other families, the age and education of the household head, and an index of household wealth. Standard errors are clustered at the strata (region) level. * Significant at 10 percent level; ** Significant at 5 percent level; *** Significant at 1 percent level.

Figure A1: Illness incidence amongst children under 5 in Dakar vs other urban areas in Senegal

Panel A. Diarrhea incidence

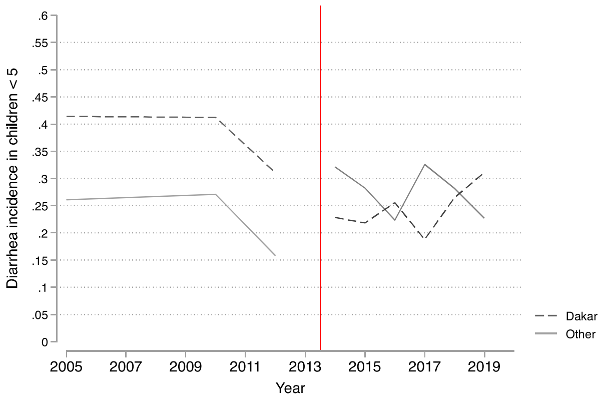

Panel B. Cough incidence

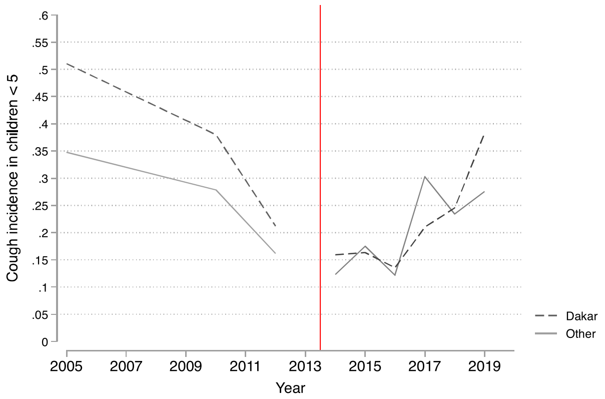

Note: Estimated weighted average values for disease incidence across years for Dakar and other cities in Senegal with at least 100k inhabitants ($N = 7$) between 2005-2019. Dependent variables are the incidence of diarrhea (Panel A) and the incidence of cough (Panel B). Controls include the number of children under 5, the household's water source, toilet type, if the toilet is shared with other families, the age and education of the household head, and an index of household wealth.
